# Concurrent stimulation and sensing in bi-directional brain interfaces: a multi-site translational experience

**DOI:** 10.1088/1741-2552/ac59a3

**Published:** 2022-03-31

**Authors:** Juan Ansó, Moaad Benjaber, Brandon Parks, Samuel Parker, Carina Renate Oehrn, Matthew Petrucci, Ro’ee Gilron, Simon Little, Robert Wilt, Helen Bronte-Stewart, Aysegul Gunduz, David Borton, Philip A Starr, Timothy Denison

**Affiliations:** 1 Department of Neurological Surgery, University of California, San Francisco, CA, United States of America; 2 MRC Brain Network Dynamics Unit, Nuffield Department of Clinical Neurosciences, University of Oxford, Oxford, United Kingdom; 3 Department of Electrical and Computer Engineering, University of Florida, Gainesville, FL, United States of America; 4 School of Engineering and Carney Institute, Brown University, Providence, RI, United States of America; 5 Department of Neurology and Neurological Sciences, Stanford University School of Medicine, Stanford, CA, United States of America; 6 Department of Neurology, University of California San Francisco, San Francisco, CA, United States of America; 7 Department of Biomedical Engineering, University of Florida, Gainesville, FL, United States of America; 8 Institute of Biomedical Engineering, Department of Engineering Science, University of Oxford, Oxford, United Kingdom; 9 Shared first author.; 10 Shared senior author.

**Keywords:** artifacts, algorithms, adaptive deep brain stimulation, closed loop, neural sensing, chronic implant, embedded

## Abstract

**Objective.:**

To provide a design analysis and guidance framework for the implementation of concurrent stimulation and sensing during adaptive deep brain stimulation (aDBS) with particular emphasis on artifact mitigations.

**Approach.:**

We defined a general architecture of feedback-enabled devices, identified key components in the signal chain which might result in unwanted artifacts and proposed methods that might ultimately enable improved aDBS therapies. We gathered data from research subjects chronically-implanted with an investigational aDBS system, Summit RC + S, to characterize and explore artifact mitigations arising from concurrent stimulation and sensing. We then used a prototype investigational implantable device, DyNeuMo, and a bench-setup that accounts for tissue–electrode properties, to confirm our observations and verify mitigations. The strategies to reduce transient stimulation artifacts and improve performance during aDBS were confirmed in a chronic implant using updated configuration settings.

**Main results.:**

We derived and validated a ‘checklist’ of configuration settings to improve system performance and areas for future device improvement. Key considerations for the configuration include (a) active instead of passive recharge, (b) sense-channel blanking in the amplifier, (c) high-pass filter settings, (d) tissue–electrode impedance mismatch management, (e) time-frequency trade-offs in the classifier, (f) algorithm blanking and transition rate limits. Without proper channel configuration, the aDBS algorithm was susceptible to limit-cycles of oscillating stimulation independent of physiological state. By applying the checklist, we could optimize each block’s performance characteristics within the overall system. With system-level optimization, a ‘fast’ aDBS prototype algorithm was demonstrated to be feasible without reentrant loops, and with noise performance suitable for subcortical brain circuits.

**Significance.:**

We present a framework to study sources and propose mitigations of artifacts in devices that provide chronic aDBS. This work highlights the trade-offs in performance as novel sensing devices translate to the clinic. Finding the appropriate balance of constraints is imperative for successful translation of aDBS therapies.

## 1. Introduction

**Figure 1.**
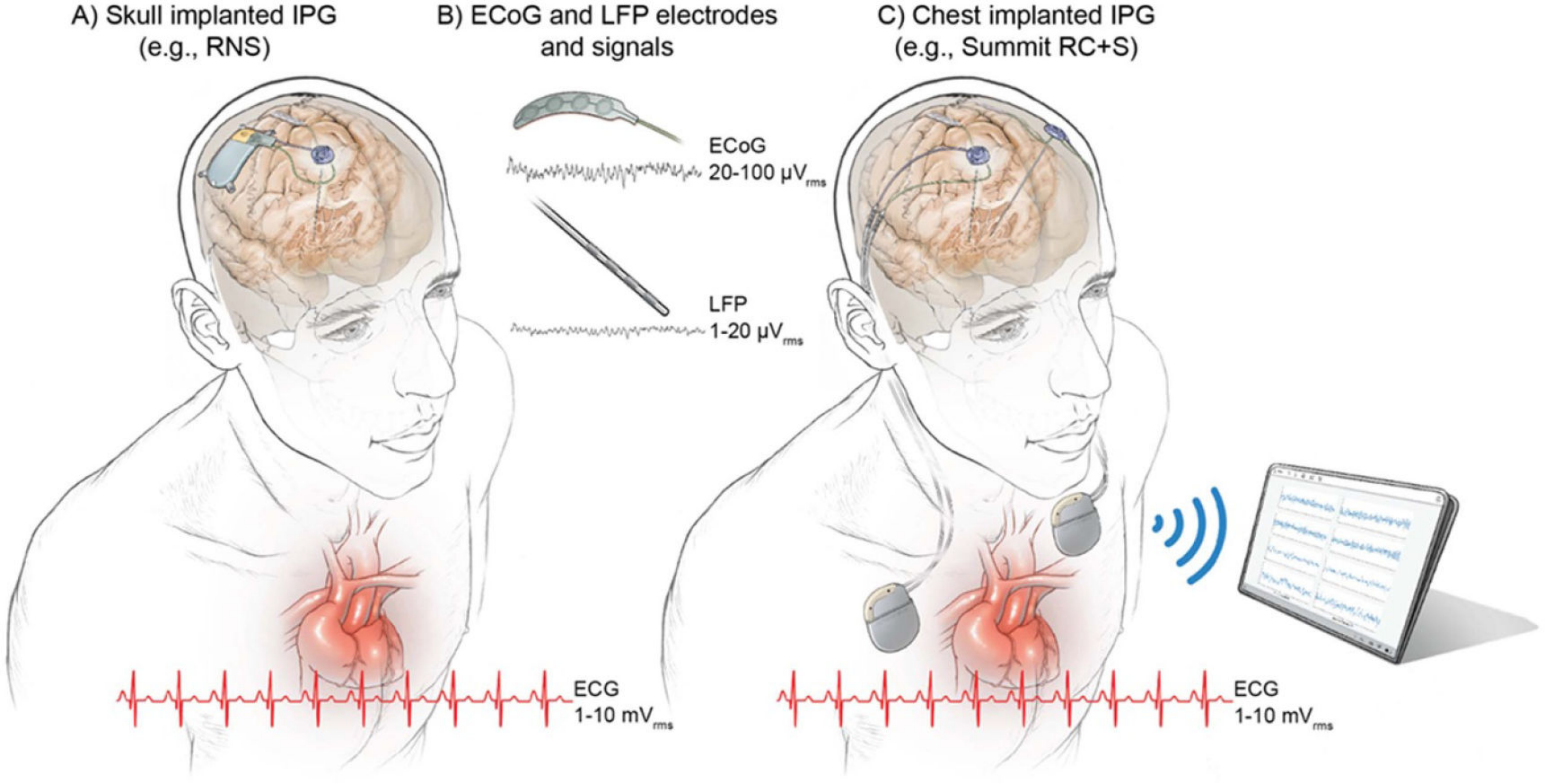
Configurations of chronic implanted sensing pulse generators and sense electrodes. (A) Example of a skull implanted internal pulse generator (IPG) (e.g. RNS, NeuroPace Inc.), (B) example of quadripolar ECoG and DBS leads and ranges of neural signals (LFPs running a few microvolts and ECoG signals ranging a few tens of microvolts). (C) Example of an aDBS system that is implanted in the cervical space (or chest cavity); in this example the Summit RC + S (Medtronic) investigational system. The heart anatomy is shown along with representation of ten cycles of the cardiac signal with amplitude range × 1000 the neural signals (millivolt instead of microvolt). Notice the close location of chest implanted IPGs (C) to the heart, which may influence leakange of ECG artifacts into the neural signal. ECG artifact is avoided in skull mounted IPGs (a).

Implantable devices for chronic neurostimulation are rapidly evolving and several now incorporate a neural sensing component as well as the capacity to utilize neural signals for rapid modification of stimulation parameters. This is often called ‘adaptive’ deep brain stimulation (aDBS) (figure 1) [1-10]. However, technical challenges still limit the clinical applicability of adaptive closed-loop deep brain stimulation (DBS) [11, 12]. The small (microvolt level) magnitude of neural signals in comparison with the size of artifacts (millivolt level) challenges signal fidelity and detection algorithm accuracy [13-19].

**Figure 2.**
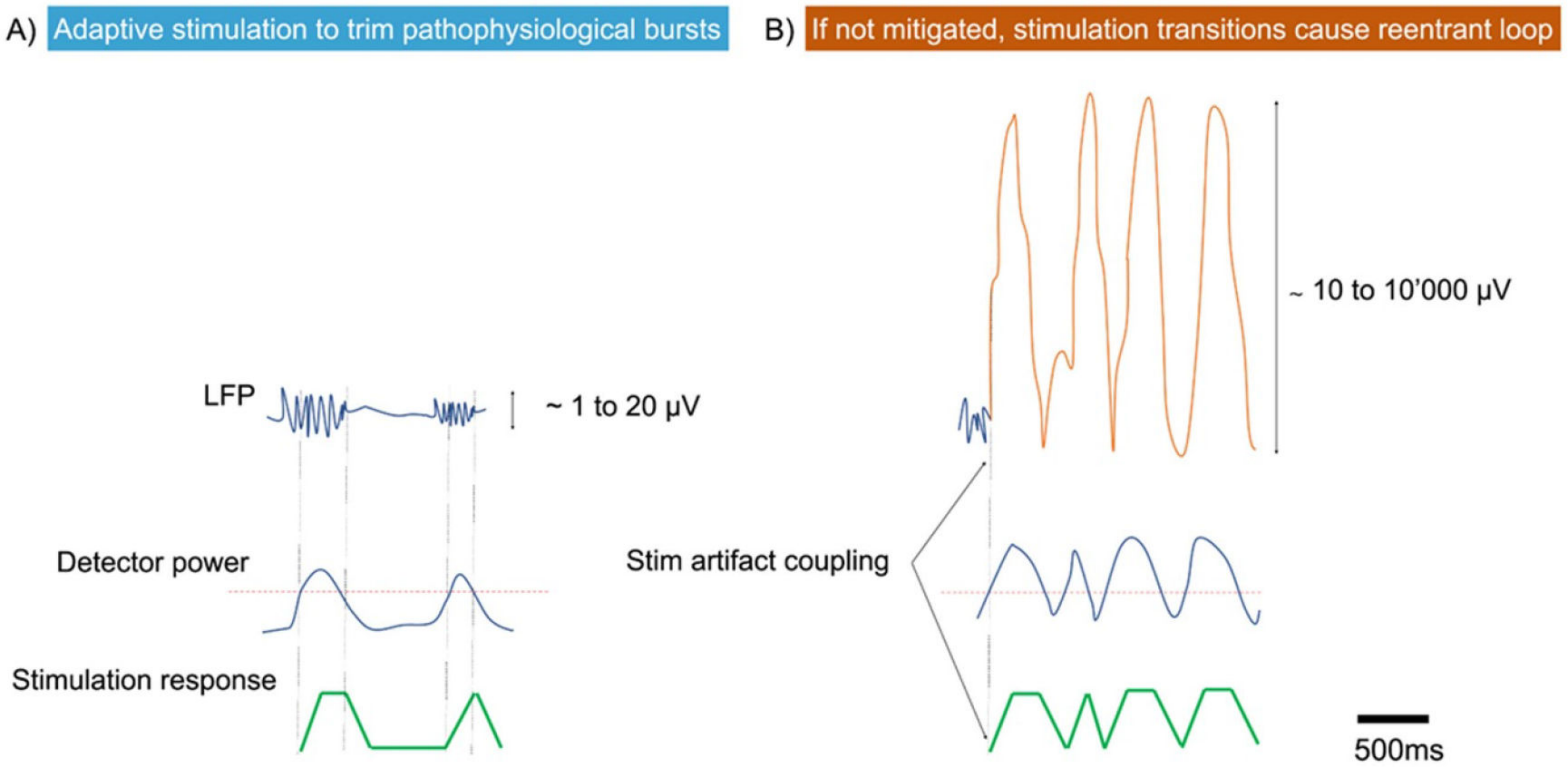
‘Ideal’ versus ‘reentrant loop’ (or ‘self-triggering’) scenarios during ‘fast’ aDBS: (A) ‘Ideal’: stimulation is triggered by an increase in amplitude or power of the predefined biomarker bandpass LFP signal. (B) ‘Reentrant loop’ or ‘self triggering’: the onset of stimulation as response to the first detection of the biomarker signal results in a transient response due to the stimulation ramp or stimulation transitions coupled in the sensed signal. The contaminated LFP signal increases detector power and, if not mitigated, may result in false-detection and the detector will go into reentrant loop or ‘self-triggered’ stimulation, not responsive to physiological changes.

While DBS has become a standard treatment for mid-stage Parkinson’s disease (PD), there is great interest in improving its efficacy using adaptive algorithms. Some of the early promising algorithms on aDBS [20-23] involved fast (~0.5 s) switching of stimulation to respond to (or to shorten) pathological bursts of oscillatory activity. Duration and amplitude of bursts in the beta frequency range (13–30 Hz) correlate with severity of rigidity and bradykinesia [24], are associated with freezing of gait [25] and are modulated by medication cycle [26]. Stimulating only at the onset of ‘pathologic’ beta bursts is technically demanding due to transient currents coupled in the sensing channel during ‘fast’ increase/decrease of stimulation amplitude [2, 27] (figure 2). Although this paradigm has shown promise in patients with externalized DBS leads, its technical viability in an embedded configuration (without externalized leads or external computation) requires further research. ‘Slower’ control algorithms that adjust stimulation amplitude on a time scale of minutes–hours [6, 28, 29] have been technically easier to implement within implanted devices but nevertheless also depend on careful management of artifacts related to sensing during stimulation.

The application of sensing and feedback control algorithms might improve other therapies. DBS in psychiatric disorders, epilepsy or chronic pain currently apply a variety of stimulation and sensing regimes [1, 8, 30-32]. For example, during ‘half duplex’ aDBS, stimulation is turned OFF during sensing, and only turned ON for a prespecified duration in response to detection of a predefined neural signal pattern. The responsive neurostimulator (RNS) (NeuroPace, Inc.) utilizes this type of sensing and stimulation paradigm, and it is approved by the US Food and Drug Administration for the treatment of some types of epilepsy [1]. While this (interrupted) sensing/stimulation paradigm may be sufficient for epilepsy and other paroxysmal disorders, it assumes we do not need to know when to turn off stim, and fixed pulse durations suffice. Our scope of application is for use cases that require real-time (short latency) tracking of biomarker fluctuations in the presence of therapeutic stimulation, which we denote as ‘full duplex’ paradigms. At this time, full-duplex communication is expected to be the method preferred for most movement disorders [6, 20, 24, 33, 34]. Some recent studies of closed-loop DBS for psychiatric disorders [35, 36] and for refractory epilepsy [37, 38] have also explored the use of full duplex paradigms. Therefore, the pathophysiology of the disease under study, such as minimum ranges of detectable neural signals and acceptable time latencies between sensing and stimulation responses set the key specifications for the device (supplementary material, table 1 available online at stacks.iop.org/JNE/19/026025/mmedia). Of note is the dependence of signal level on location of the electrode, even within the same disease state. In general, cortical signals (20–100 *μ*Vrms) are a factor of ten larger than subcortical signals (1–20 *μ*Vrms) from the basal ganglia or thalamus (figure 1(b)).

**Figure 3.**
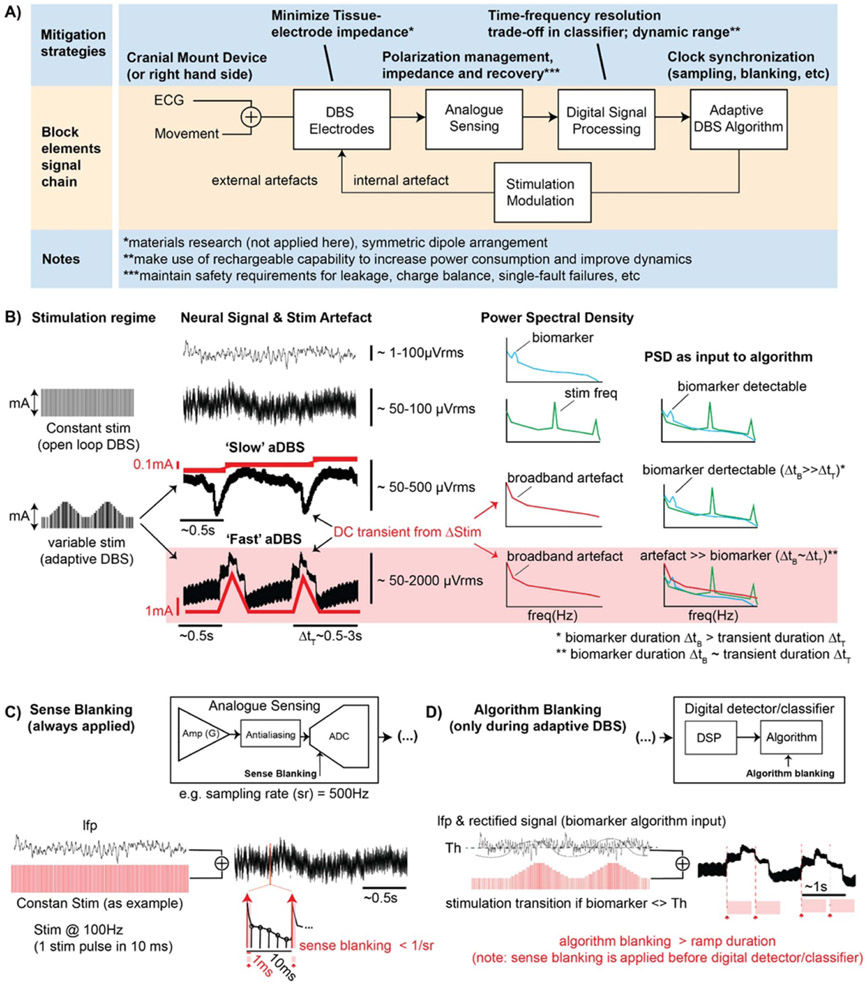
Signal chain diagram and summary of mitigation strategies of a generic aDBS system. (A) In the top row (light blue), proposed mitigation strategies to artifact susceptibility through signal chain. In the middle row (orange) the block diagram of key signal chain elements, with possible artifacts due to external triggers, such as ECG signal and movement artifacts; or due to stimulation. (B) Stimulation regimes: constant stimulation (e.g. 2 mA and 130 Hz) or varying stimulation following biomarker variations (aDBS). Rate of change in amplitude is defined by the stimulation ramp rate, with ‘slow’ ramps ranging 0.1–1 mA s^−1^ and ‘fast’ ramps from 1 to 10 mA s^−1^. Variable stimulation amplitude (red time-varying traces) results in transient step responses contaminating the LFP in the neighborhood of the stimulation electrode (black time-varying traces). Examples of biomarker signature (blue), stimulation artifact (green), and broadband artifact due to stimulation ramp (red) for a frequency-based detector/classifier. (C) Sense blanking is part of the analog chain and is always synchronously applied following the stimulation clock for a duration in the order of a few milliseconds (≫duration of stimulation pulse). By blanking the analog sense channel for a duration of ~ms the artifact in the sense channel due to the stimulation pulse can be rejected while the LFP signal is not missed. Typically the sense blanking duration falls in the order of the time between consecutive samples (1/sampling rate). Sense blanking is applied continuously, during both constant stimulation and adaptive stimulation. (D) Algorithm blanking is part of the digital chain and is synchronously applied to an algorithm detection event (e.g. biomarker input crosses predefined threshold Th). Algorithm blanking is defined with a certain duration based on biomarker physiology time/frequency dynamics and device specifications. By applying algorithm blanking to the input signal of the detector, the aDBS algorithm is blanked for that duration (hundred milliseconds to few seconds; typically at least for the duration of the stimulation ramp). By selecting appropriate algorithm blanking values, stimulation transition artifacts can be mitigated and detector performance improved. However, setting algorithm blanking parameters may be challenging for ‘fast’ aDBS because the duration of stimulation transients and time/frequency dynamics of the biomarker (e.g. beta band) converge at time scales of 1 s or less.

Bi-directional neural interfaces that support sensing and stimulation have common building blocks, as illustrated in figure 3. An electrode at the neural interface provides access to the biological environment and transduces between electron-based current flow in the tissue and ionic-based current flow in the device. The electrode properties set many key constraints for sensing and stimulation, which include impedance, impedance mismatch between sensing electrodes, polarization, and safe charge transfer (see supplementary material, background section). For sensing physiological signals, a low noise preamplifier will condition the signal and typically extract a differential measurement between an electrode and a reference; the reference can be far-field, but is typically another local electrode in DBS applications to improve specificity to the signal of interest, or to reduce stimulation artifacts by recording symmetrically around the stimulating contact. The amplified signal is then passed to an analog-to-digital converter, and the signal is processed with digital filters and classifiers (see supplementary material, figure S1).

Typical classifiers include spectral power in discrete bands related to a physiological signature of the disease process, which can be estimated with digital bandpass filters or fast-Fourier-transforms (FFT). The adaptive algorithm sets the control policy for adjusting the system, and in many current systems [2, 6, 23, 27] simply applies changes to stimulation amplitude based on a classification such as threshold detection. More nuanced adaptive algorithms using proportional-integral control or multiple thresholds are also under investigation [39, 40]. The control policy of the algorithm then adjusts the stimulator parameters, and the stimulation current is delivered through an electrode pair. This model of the bi-directional interface highlights the key constraints and considerations for optimization of the signal chain for concurrent sensing and stimulation. We define the general architecture of feedback-enabled devices (figure 3), propose key components in the signal chain which might result in unwanted artifacts and propose methods that might ultimately enable improved aDBS therapies (table 1).

Mitigation can be achieved by a combination of optimized parameters: the analog sensing chain (analog filters, amplifier common mode rejection and ‘sense blanking’), the digital signal processing (time and frequency resolution filtering trade-offs and the detector properties of the adaptive algorithm, e.g. ‘algorithm blanking’). Rapidly switching stimulation ON–OFF (extreme case: open loop mA → 0 mA), if not mitigated may result in broadband spectral artifacts and limit performance of aDBS algorithms. As examples, the power spectrum of the sensed neural signal (and artifact) are represented in the third column of figure 3(B) for ‘slow’ and ‘fast’ aDBS regimes. The effect of the broadband artifact on the spectrum of the input detector signal is shown in the most right column of figure 3(B). In the most extreme scenario, ‘fast’ ON–OFF stimulation transitions, an algorithm blanking of the order of the biomarker’s time dynamics (e.g. beta bursts, 0.5 to ~1 s) may not be sufficient to mitigate transient effects resulting in a classifier reentrant loop with the risk of missing pathophysiological bursts.

The relative topology of stimulation and sense electrodes influences the magnitude of commonmode (CM) artifacts versus differential sense signal (common mode refers to interference coupled in the sense channel, such as stimulation, electrocardiogram (ECG) or movement, with approximately equal contribution in each of the sense electrodes). Using a symmetric sensing configuration around the stimulation electrode leverages the CM rejection of the differential amplifier with the neural signal captured as a differential dipole or local field potential (LFP or subcortical neural signal measured via two neighboring electrodes). This optimized symmetric sense/stimulation configuration is sensitive to impedance imbalances between electrodes and thus may still capture CM artifacts. To further reduce common mode artifacts and their impact during aDBS, sense electrodes may be placed farther away from the stimulation dipole (e.g. sensing in the motor cortex while stimulating subcortically). Proof of concept implementations of embedded aDBS with a far-field sensing topology have recently been demonstrated for ‘slow’ [6, 41] and ‘fast’ [34] paradigms. Furthermore, the common mode stimulation artifact in the LFP can be used to drive another implantable device on the contralateral hemisphere [42].

**Figure 4.**
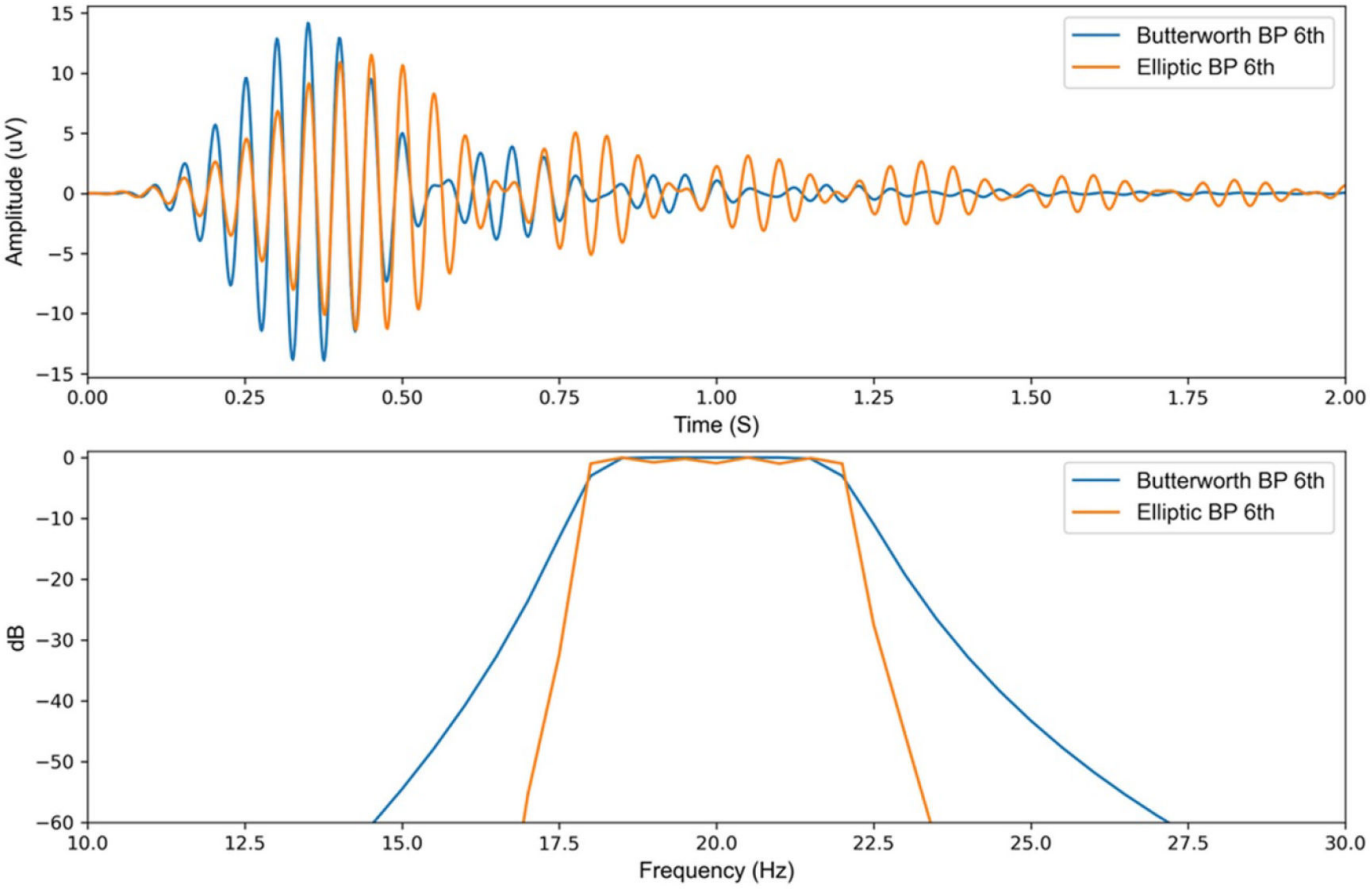
Comparison between a 6th order Butterworth and elliptic bandpass filters, both with a bandwidth of 18–22 Hz (A) 1 mV Impulse response of the two bandpass filters to illustrate the difference in settling time and ringing of the filters in the time domain. (B) Frequency response of the two bandpass filter to illustrate the difference in frequency specificity of the two filters.

The design of the digital signal processing chain of the aDBS algorithm also affects system performance. aDBS algorithms that use power-threshold detection regimes require the use of a bandpass filter, either in the frequency (FFT) or in the time domain, to select the frequency of interest. When designing such a filter, there is a tradeoff between frequency specificity and time-domain operation. Figure 4 illustrates the time and frequency response difference for two types of digital filters. In the event of any sudden large changes in the input signal (e.g. stimulation transient response), the Butterworth filter which has less frequency specificity, results in a shorter ringing period. While an Elliptic filter with higher frequency specificity, results in a longer ringing duration in the time domain.

In this paper, we provide a framework to describe ‘real-world’ neural signal artifacts in chronic implanted sensing pulse generators in a translational setting (human research). We aim to characterize technical performance of neural interfaces to mitigate these artifactual signals and assess performance of ‘fast’ adaptive closed-loop DBS algorithms. We provide a generic bench model that can help compare different approaches and that mimics real-world performance to allow rapid prototyping of algorithms prior to human testing. We use these methods to define a checklist of considerations for optimizing the signal chain and validate its utility in a chronic implant.

### 1.1. Characterization test methods (chronic, *in vivo* devices)

In a series of movement disorder patients implanted with an investigational sensing pulse generator, the Summit RC + S (Medtronic) [2, 6, 37], neural field potentials were recorded during supervised visits with a variety of stimulation settings (supplementary material, table S2, NCT02649166/IRB201501021, NCT04043403/IRB52548, NCT03582891/IRB182 4454, IDE #180 097). All patients had DBS open loop DBS settings clinically optimized based on standard monopolar review during the first month after implantation. Sensing topology in the subcortical DBS lead was a symmetric dipole (sandwiching) around the stimulation electrode (figure 6(a)) with monopolar stimulation referenced to the internal neurostimulator. In a subset of subjects (all except subject ID4), two additional sensing channels were added to capture electrocortical (ECoG) signals from the somatosensory and motor cortical areas. We studied three possible types of artifacts during concurrent stimulation and sensing: (a) ECG cardiac signal (Stim ON ⩾ 0 mA), (b) transient responses in the sense channel for different stimulation ramping speeds (0.5–4 mA s^−1^), (c) transient responses during a ‘fast’ aDBS for beta suppression. During (a) and (b) RC + S was configured in ‘distributed’ mode and without adaptive stimulation since we were only interested in artifact signals in the sense channel due to concurrent sense and stimulation. In (c), the RC + S was configured in ‘embedded’ mode and with adaptive stimulation to follow biomarker power threshold transitions.

#### 1.1.1. Tissue electrode impedance

To study the degree of impedance mismatch between sense electrodes at the input of the analogue signal chain, lead impedances were recorded in a subset of subjects at intermittent time points from implantation date and beyond one year after initiation of DBS. The impedance values were recorded by the sensing IPG system (Summit RC + S, Medtronic) when interrogating it with the command ‘check lead integrity’. Monopolar, single contact electrode impedances (e.g. C1 or electrode contact 1) were measured referenced to the IPG case. Bipolar impedances were defined as those created by pairing subcortical electrodes providing a symmetric sense dipole around a monopolar stimulation contact (i.e. C0–C2 and C1–C3). Impedance mismatch of subcortical electrode pairs was defined as the absolute difference of the impedance of each contact divided by the mean value between them.

#### 1.1.2. Cardiac artifact in deep leads with stimulation ON

Subcortical stimulation and concurrent subcortical and cortical sensing was performed using a four-electrode subcortical lead (Medtronic model 3387) targeted at the ventralis intermedius nucleus region of the thalamus (VIM). Monopolar stimulation was delivered between electrode contact 2 and the neurostimulator (Summit RC + S, Medtronic) located in the patient’s left chest cavity. Three bipolar field potential channels were recorded during stimulation: (Ch1) Subcortical sandwich configuration (C1, C3) around the stimulation contact (C2). (Ch2/Ch3) Cortical field potentials were recorded from the primary motor and somatosensory cortex using quadripolar cortical paddles (Medtronic model 0913025). Stimulation settings included 0 mA (stimulation engine on but not delivering stimulation) and 1.0 mA (clinical stimulation level), with a 90 *μ*s pulse width and a 135 Hz stimulating frequency. Two separate waveforms were tested: passive recharge, which involves a square stimulation pulse followed by an extended recovery in the opposite polarity using passive circuits for charge balance; and active recharge, which sends two symmetric pulses sequentially for both stimulation and recovery, allowing for charge balance in a much shorter time frame. The following settings were used for recording from the three channels: sampling rate 500 Hz, high pass filter at 0.85 Hz and low pass filters at 100 and 100 Hz, with a sense blanking value of 2.5 ms.

#### 1.1.3. Ramp impacts on transient step responses

Stimulation was delivered subcortically while ramping artifacts were tested in both subcortical and cortical channels using a variety of waveform and sense blanking values. Two waveforms were tested; active and passive recharge. Sense blanking, with the Summit RC + S system, is a sliding value that can be set to values ranging from 0.335 up to 2.505 ms (note that ‘sense blanking’ is a short blanking period at the analog amplifier chain synchronized with the stimulation pulse and prior to the ‘algorithm blanking’, which is of a much larger duration (>50 ms) and takes place as part of the detector/classifier algorithm, see supplementary material figure S1). Here we tested the default ‘amplifier sense blanking’ value of 0.335 ms, as well as 1.005 ms and the maximum value possible of 2.505 ms. During this recording session, the ‘operative mode’ on the neurostimulator was activated. Different from ‘adaptive mode’, the ‘operative mode’ allows for manually switching between states.

#### 1.1.4. Duration of stimulation ramp transient step response

We studied the duration of the transient step response during and after stimulation ramping. We used ramping rate datasets ranging from 0.5 to 4.0 mA s^−1^, collected from patients with the RC + S (subject IDs 1,2 see supplementary material, table S2). We characterized the transient response as a function of the ramp rate, for two different stimulation amplitude excursions (0–1 mA and 0–1.6 mA). Sensing settings were the same as previously described. Constant stimulation settings are 90 *μ*s pulse widths and 135 Hz stimulation frequency; the amplitude ranges from 0 mA up to the clinical stimulation amplitude for each subject (1 or 1.6 mA), with the ramp rate varying based on patient comfort (paresthesia occurring during rapid ramp rates)t. The transient response duration is defined from the time ramping begins until the signal returns to approximately the same baseline value as prior to ramping (assuming return to baseline as the optimal for best signal characterization).

#### 1.1.5. Fast aDBS

In supervised sessions of PD subjects (ID8,10,12) receiving open loop DBS for ~1 year with bilateral Summit RC + S implants, we tested ‘fast’ aDBS at highest tolerable (no paresthesia) ramp rates (0 mA to open loop stim level in 200–300 ms). All subjects had undergone bilateral placement of cylindrical quadripolar deep brain stimulator leads into either STN (subthalamic nucleus) (Medtronic model 3389) or GP (globus pallidus) (Medtronic model 3387) and bilateral placement of paddle-type quadripolar cortical paddles into the subdural space over the motor cortex (Medtronic model 0913025) [6]. Monopolar stimulation was delivered between deep electrode contacts 1 or 2 and the IPG. Three bipolar field potential channels were recorded during stimulation: (Ch1) Subcortical sandwich configuration (C0, C2) or (C1, C3) around the stimulation contact C1 or C2 respectively. (Ch2/Ch3) Cortical field potentials recorded from primary motor and somatosensory cortex (note: here we tested ‘fast aDBS’ using subcortical concurrent sense/stimulation). Stimulation settings included 0 mA (low target) and the clinical stimulation level (different for each subject), with a 60 *μ*s (in STN) or 90 *μ*s (in GP) pulse width and a 130 Hz (STN) or 150 Hz (GP) stimulating frequency. The following settings were used for recording from the three field potential channels previously mentioned: sampling rate 500 Hz, high pass 0.85 Hz and low pass 100 Hz, sense blanking of 0.5 or 1 ms and ‘sense-friendly’ stimulation frequency (closest stimulation rate to the desired frequency value that is optimized within the RC + S sensing circuitry to couple minimal stimulation artifact).

The RC + S was configured for embedded (on device) aDBS with the following settings: sampling rate 500 Hz, FFT size 256, FFT update rate 100 ms, FFT overlap 80%. Subject specific beta biomarker frequency bands were defined as the area within the canonical beta band with the largest peak while turning DBS therapy OFF during 1–2 min. A single algorithm detector threshold was defined as ~50% of the biomarker power range during stimulation OFF. To interface with the RC + S device during aDBS parameter exploration and algorithm testing we used our open-source Research Facing App software^11^. Stimulation ramps were defined from 0 to maximum stimulation (open loop clinical stimulation) within a time window of 200–300 ms, after verifying no paresthesia reported by patient.

For off-line data visualization of the RC + S raw data we used our open-source MATLAB library [43]. After ingesting the data, the raw time domain signal was bandpass filtered (Butter and Hilbert functions, MATLAB) and centered at the predefined (experimental) beta frequency band. Power spectrum density (PSD) of representative 2 s time domain segments were computed using pwelch (MATLAB) with a window length of 400 ms and 50% overlap. A resulting averaged PSD over the 2 s window was plotted.

### 1.2. Characterization test results (chronic, *in vivo* devices)

#### 1.2.1. Impedance mismatch in subcortical sensing electrodes

**Figure 5.**
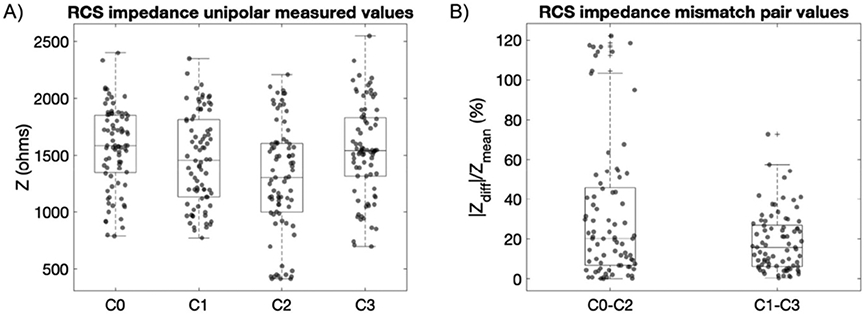
Subcortical impedances measured with the RC + S system in a group of movement disorders patients (7 STN, 4 GP, 2 dystonia) represented as absolute unipolar and percentage difference mismatch between paired electrodes. (A) Distribution of single measurement points of each subcortical electrode contact referenced to the IPG case, with a range of variability from 500 to 2500 ohms. (B) Impedance mismatch of a subcortical electrode pair defined as the absolute difference of the impedance of each contact divided by the mean value between them. Electrode pairs are defined as those capable of providing a symmetric sense dipole around a monopolar stimulation contact. With a quadripolar subcortical lead, this leads to stimulation contacts C1 or C2 with subsequently symmetric sense pair electrodes C0–C2 or C1–C3. By pairing electrodes in this manner, calculated impedance mismatches were 20.2% (75th percentile 45.7%) for C0–C2 and 15.8% (75th percentile 26.9%) for C1–C3. Note that the outliers observed in C0-C2 (panel (B)) could be due the large range of variability between C0 and C2 (panel (A)). This large variability can be expected due to: (a) the different target electrode locations (STN or GP), (b) the use of different electrode lead models (geometry of STN electrode lead differs from GP lead), and (c) the impedance variability of intrinsic anatomical target regions due to different brain conductivity media (in the GP contact C2 is placed at the intersection of the GPe and GPi (gray and white matter), see supplementary material figure S1).

A total of 89 impedance measurement points were collected in 14 different patients. After excluding outliers (8/89 points) due to technical malfunctioning in the IPG-lead connection (high impedance ~50 Kohm; issue in one side of two patients, resolved on later cases by adding medical adhesive to the IPG-lead connector), a total of 81 points were part of the analysis. By pairing electrodes to allow for optimal subcortical aDBS configuration, impedance mismatches ranged 5%–45% (25th–75th percentile) with median values ranging 15%–20% (see figure 5, and similar figure from ET subjects in supplementary material figure S2).

#### 1.2.2. Active recharge reduces cardiac artifact from deep leads

**Figure 6.**
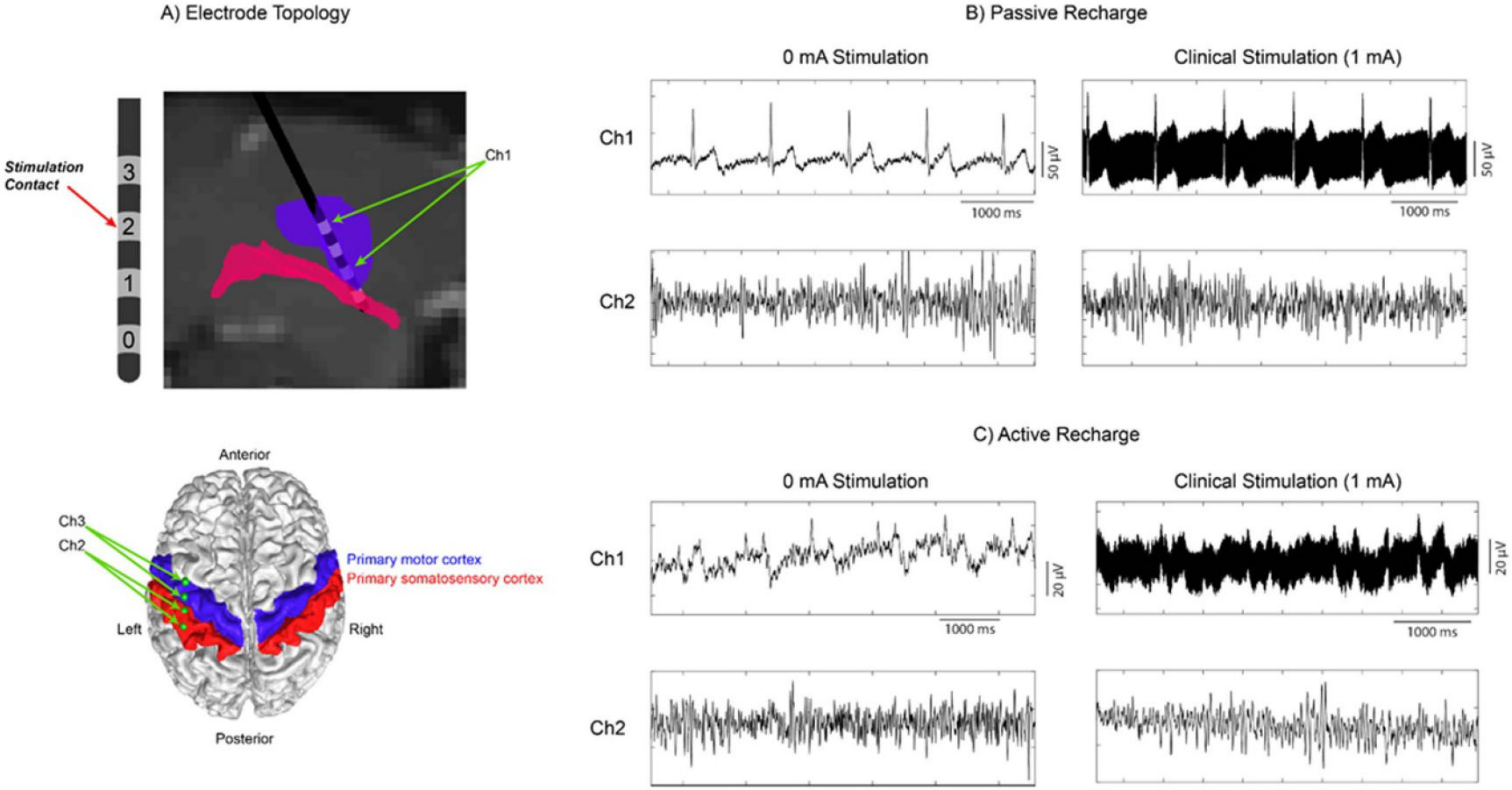
ECG and high frequency stimulation artifacts during concurrent stimulation and sensing with the bidirectional neural interface (Sumit RC + S, Medtronic) in a patient with ET. (a) Electrode placement within the VIM, and cortical strip placement over the primary motor/somatosensory cortices (Ch1 subcortical channel and Ch2, Ch3 cortical channels). (b) Recording during passive recharge at 0 mA and clinical stimulation levels, at Ch1 (subcortical channel) and Ch2 (cortical channel). Passive recharge utilizes a square pulse followed by a long-term small-amplitude charge in the opposite polarity. (c) Recording during active recharge using the same amplitudes and channels as (b). Active recharge sends sequential symmetric square pulses with opposite polarities, balancing the charge in a shorter time period.

Stimulation with passive recharge results in larger ECG residual artifact compared to active recharge at the sensing electrodes proximal to the stimulation electrode (figure 6). ECG artifact was seen as long as the stimulation engine is turned on even with an amplitude of 0 mA. During clinical stimulation, a stimulation artifact at 135 Hz is seen superimposed on the underlying signal, with the cardiac artifact continuing to appear during passive recharge. Active recharge not only reduced the cardiac artifact, but also reduced the total power of the stimulation artifact. The ECG artifact was clearly seen in two essential tremor (ET) subjects during passive recharge, with one representative dataset shown here. There was a significant reduction in the artifact during active recharge (figure 6(b) versus figure 6(c)). All subsequent subjects were recorded during active recharge at all times, which makes it difficult to say whether or not the temporal fluctuations seen are ECG or not. No artifact was observed in any configuration when viewing the cortical channel (Ch2).

#### 1.2.3. Sense blanking and active recharge reduces DBS artifact

**Figure 7.**
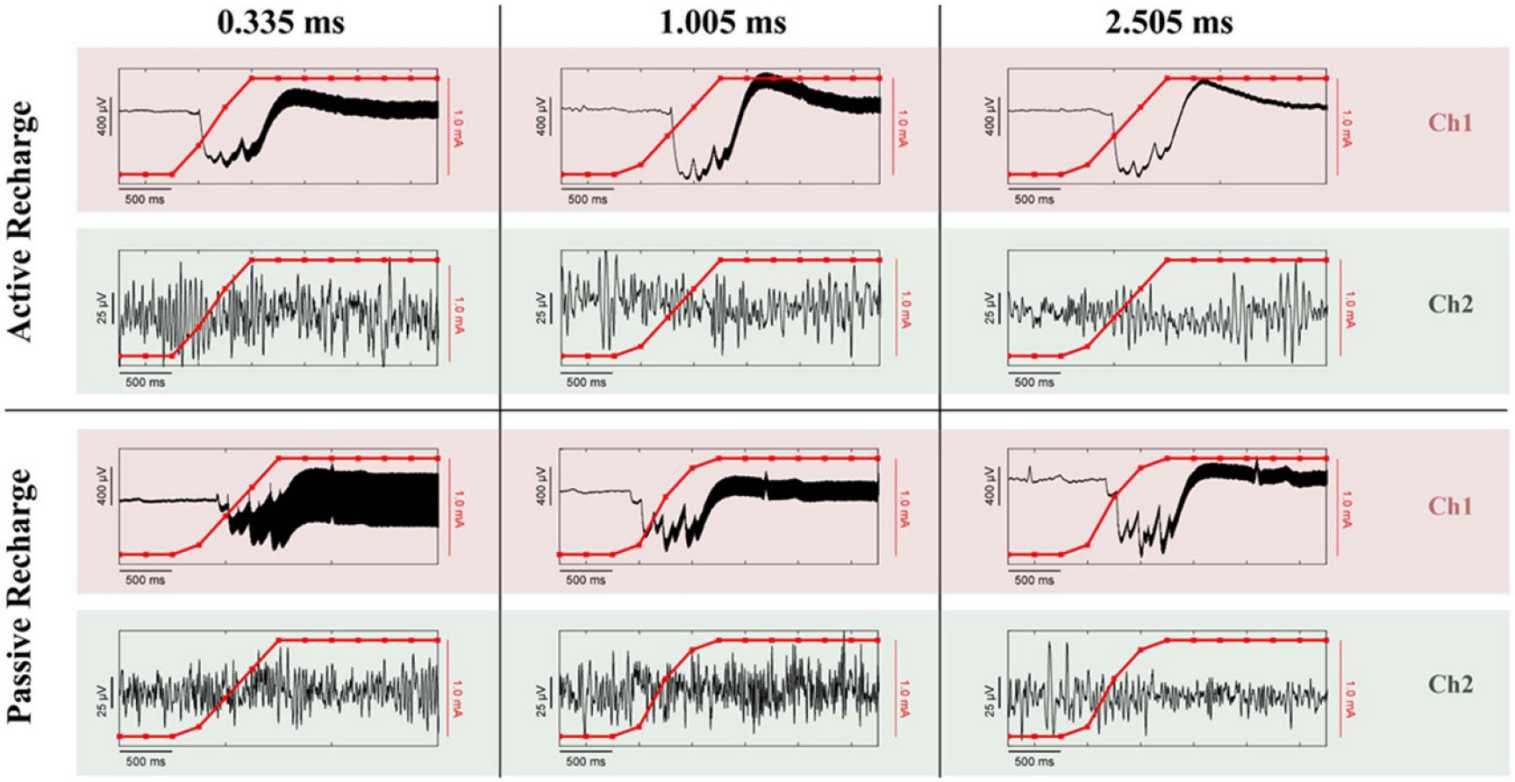
Comparison of the effect of waveform and sense blanking values on DC transient artifacts during ramping and high frequency stimulation artifacts. Every combination of waveform/sense blanking values contains both a depth channel (Ch1) and a cortical channel (Ch2) for comparison of near-field and far-field recording contacts, respectively. Each panel depicts the time-domain signal as a black line, and the current amplitude of the device while ramping up in red.

Ramping the stimulation (figure 7) while sensing results in both a high frequency stimulation artifact and a direct current (DC) transient artifact at the near-field sense electrodes (Ch1), while the same artifacts do not appear at the far-field electrodes (Ch2). Note that the electrode montage placement is the same as figure 6(a). All permutations shown were recorded from the same patient on the same day, with the only differences being the sense blanking value (0.335/1.005/2.505 ms) and the stimulation waveform (active/passive recharge). Higher sense blanking values reduced the magnitude of the high-frequency stimulation artifact in Ch1, but the DC transient artifact reached approximately the same value no matter the configuration. Active recharge was better than passive recharge at each sense blanking value, with the added benefit that while using active recharge the ECG artifact seen during passive recharge is also reduced (also seen in figure 6(b)).

#### 1.2.4. Duration of stimulation ramp transient step response

**Figure 8.**
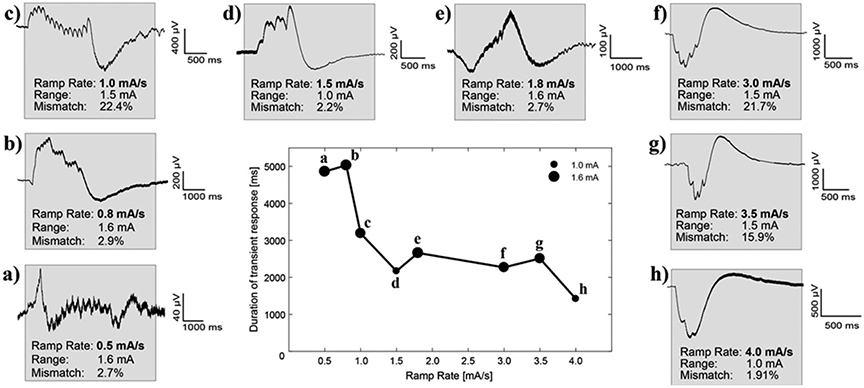
Duration of transient response as a function of ramp rate and amplitude range from *in vivo* testing (subjects ID1,2, supplementary materials table S2). The center panel shows the total time from the start of the ramping period until the signal returns to a steady state. The range of the amplitude (from the minimum value to the maximum value) is denoted by the size of the marker. (A)–(H) Sub-panels surrounding the central panel are ordered based on their ramp rate. The gray box highlights when the ramping starts, on the left edge, and when the signal returns to approximately steady state, on the right edge. Within each sub-panel is displayed the ramp rate of the run, the total range the amplitude oscillates between, and the measured impedance mismatch between the bipolar recording channels as a percentage.

Duration of the transient response was inversely correlated with both ramp rate and amplitude range, where a slower ramp rate and a large amplitude range led to the longest transient step response (figure 8). Each waveform seen in figure 8 is unique, with a convergence on the general shape occurring once the ramp rate exceeds 3.0 mA s^−1^. The impedance mismatch of each recording is recorded within each subpanel. The magnitude of the impedance mismatch influenced the magnitude of the transient response (see *y*-scale relative differences of cases with similar ramp rates, e.g. panels (a)–(c) or panels (f)–(h)). The duration of the transient response was not found to correlate with impedance mismatch (i.e. dominated by time properties of high-pass filter at the input of analog chain). Figures 8(d) and (h) both show shorter duration of the transient response times (or relaxation time) than the surrounding ramp rates, potentially due to the smaller range that the amplitude has to transition across (0–1 mA instead of 0–1.6 mA). Here we show a mix of both rising and falling ramp rates, with no differences found in the transient response timing between comparable rise/fall ramp speeds, allowing for comparison between the two directionalities. At even slower ramp rates (0.1 mA s^−1^) the ramping artifact (DC transient) occurs once within the 1 s time interval between stimulation amplitude steps of 0.1 mA (supplementary material, figures S4). The time-domain signal is returning to baseline within 0.5 s, with the spectral-domain showing broadband power increases during each ramping event. As the stimulation amplitude increases (relative change is constant 0.1 mA), the broadband power is diminishing; while still present, it is not as visually distinctive at higher amplitudes.

#### 1.2.5. Transient response during ‘fast’ adaptive

**Figure 9.**
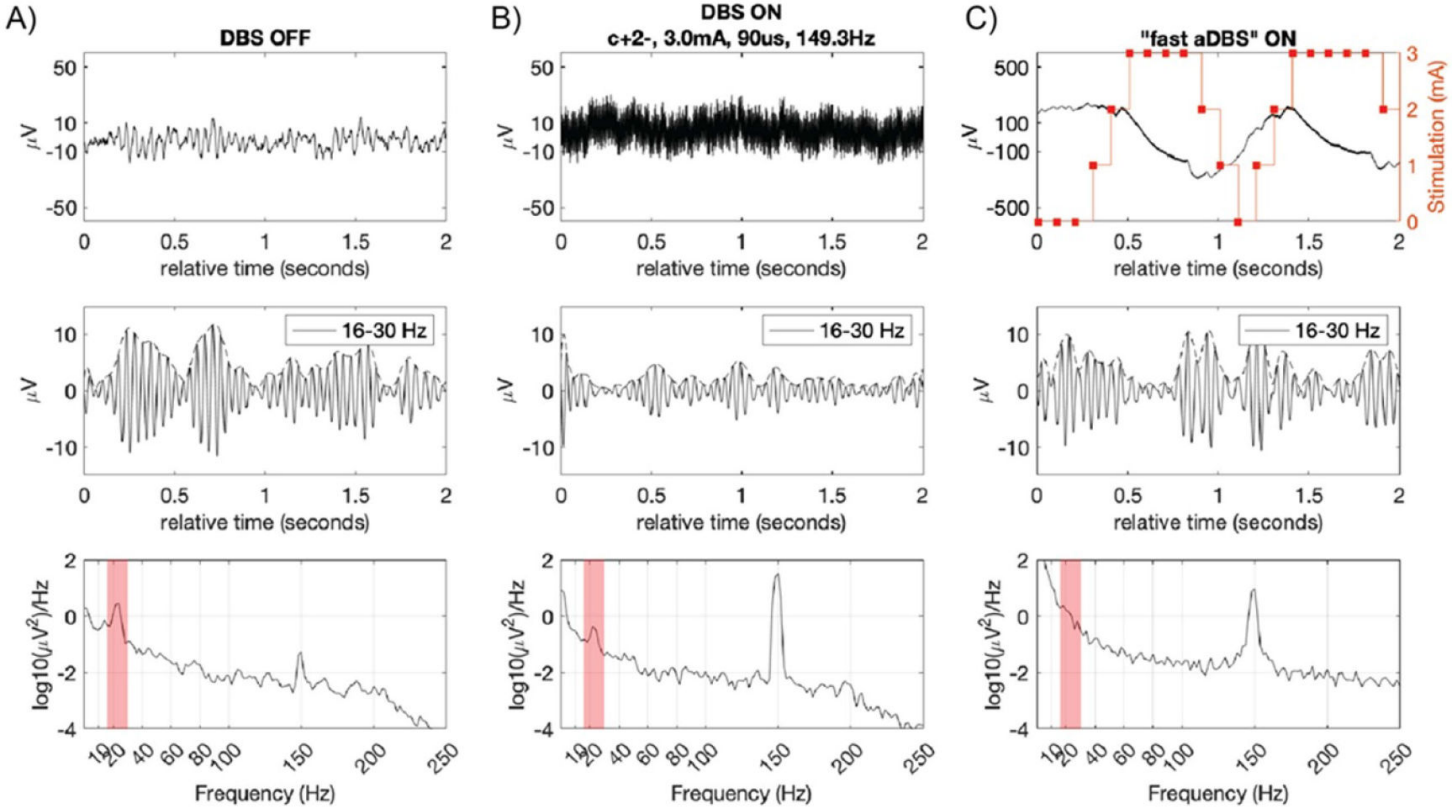
Step response artifact on LFP signal during ‘fast’ stim ramping at 10 mA s^−1^. A 2 s segment of subcortical LFP recorded from pallidum of a PD patient for three different DBS settings: (A) DBS OFF, (B) DBS ON, and (C): ‘fast aDBS’. For each DBS state (column), the top row is the LFP time domain, middle row the band pass filtered signal and the bottom row is the PSD of the 2 s segment. Sandwiched sense configuration (C1 and C3 around stim contact C2). Stimulation 0–3 mA in 300 ms (ramp up = ramp down), 150 Hz frequency, 90 us. (A) DBS OFF: no neural stimulation which results in maximal amplitude of biomarker oscillation, ~10 microvolts peak (top (A) panel). (B) DBS ON: therapeutic open-loop stimulation (cathodic monopolar stim in C2 relative to IPG in chest, 3 mA, 149.3 Hz, 90 *μ*s). (C) ‘Fast’ aDBS algorithm: top panel shows a time segment of raw LFP signal aligned with a time-varying stimulation amplitude (stimulation ramp 0–3 mA and 3–0 mA in 300 ms).

DBS A representative case of DBS OFF, DBS ON and ‘fast’ aDBS is shown in figure 9. In this subject, a reduction of beta band activity in the frequency range 16–30 Hz when turning DBS ON was found (figure 9(b)). When turning DBS ON, high frequency stimulation artifacts appeared in the raw time domain signal. By turning DBS ON a reduction of amplitude of bursts (figure 9(b) middle panel) and biomarker power band (figure 9(b) bottom panel) was observed. During ‘fast aDBS’ testing, the detector power band channel oscillated at the rate of consecutive stimulation ramps (re-entrant loop or self-triggering). This was consistent among all subjects tested with stimulation amplitude going from 0 mA to constant DBS therapy value. The stimulation ramp created a reentrant transient, overlapping the band of interest, greater than 50 *μ*V (>a factor of 10 of the magnitude of the physiological oscillation). The large transient coupled to the detection algorithm after FFT resulted in detected bursts that were confounded with physiological bursts. The confounding bursts were observed during the off-line analysis by applying the band-pass filters to the raw LFP signal (see middle panels figure 9(c)).

The power spectrum shows an increase of baseline power, which is most significant in the low frequency range of the spectrum (<30 Hz), going above the expected biomarker power (bottom panels figures 9(a)-(c)). For mitigation of the re-entrant loop issue, a detector blanking in the classification algorithm of 700 ms or higher was used, which resulted in the patient not being sufficiently stimulated at the appearance of a next pathophysiological burst. Additional examples showing how the re-entrant loop affected the aDBS algorithm can be found in supplementary materials figure S4. To verify the sources of the stimulation transients in the signal chain we turned to benchtop testing.

### 1.3. Verification: simulation and on-bench assessment of transient response during stimulation transitions

#### 1.3.1. Simulation and benchtop methods

*In-vivo* measurements in humans can limit the degrees of freedom to exploring design sensitivity and relative trade-offs. In addition, we wished to identify fundamental issues for artifact resolution, versus assessing the issues arising from a specific device design. This motivated the use of computational circuit simulations and benchtop experiments, as well as relative comparisons with another investigational device in development (Picostim-DyNeuMo-2) [5, 7] with modified approaches to implementing the canonical signal chain. To replicate the slow transient response measured in patients implanted with Summit RC + S during ON–OFF stimulation regime, a resistive and capacitive (RC-R) tissue electrode star load model was proposed. The RC-R tissue electrode interface model is based on the architectures shown in the literature by [27, 44-46]. This test setup is a simple way to mimic the interaction network of the sensing electrodes with stimulation electrodes in monopolar stimulation mode in the sandwich configuration usually used with patients.

##### (a) Electrical simulation

An electric circuit simulation was performed of the different capacitor mismatch values in the tissue–electrode star network using LTSpice XVII (Analog Devices, Wilmington MA, USA). We aimed to show the effect of switching stimulation ON–OFF on the sensing channels for the DyNeuMo-2 and RC + S sensing circuits, and how these results compared to the data recorded from patients implanted with the RC + S. The spice simulation of the RC + S and DyNeuMo-2 front-end sensing circuits was performed with the assumption of an ideal amplifier for both devices. The same values of tissue electrode interface impedances were used to test the sensing circuits of the two devices. From the simulations, a range of typical tissue–electrode interface capacitor values were identified (660 nF–2.2 *μ*F). These capacitor values match the values reported from DBS implanted electrodes [45, 47, 48].

##### (b) Benchtop testing

To test and validate the results from the simulations, a benchtop setup was created and attached to one of the devices, the DyNeuMo-2. The details on the benchtop model, hardware setup design and specification see supplementary material, *Benchtop Model and Setup*, figures S5-S7 and table S3. The benchtop test used the RC-R tissue electrode interface network and values derived from the Spice simulation. During the benchtop tests we assessed the DyNeuMo-2 sensing circuit response to capacitance mismatch in the tissue electrode interface, and how this would affect the system’s ability to perform ‘fast’ adaptive algorithms. To test a standardized worst-case scenario of the stimulation ON–OFF transients, we set the stimulation to have the same configuration throughout all the tests (3 mA active recharge stim, at 125 Hz in monopolar setup (electrode E3 back to case), and with stimulation ramping disabled). In most patients ramping is required for the stimulation setup, to avoid any paresthesia. However, as we focused on the worst-case scenario, we chose the instant switching of stimulation for a set period of time (1.6 s ON, 2.4 s OFF) throughout the tests.

##### (c) Saline testing

Saline solution usually has a homogeneous impedance throughout, so it is difficult to replicate and control the impedance mismatch as in the RC-R benchtop test. Therefore, we proposed to test, (a) the 0.9% saline baseline impedance mismatch, (b) the worst-case scenarios of ⩾100% capacitor impedance mismatch in saline. The ⩾100% capacitance impedance mismatch was achieved by shorting electrodes E1–E2 as the positive amplifier sense (+) input and using E4 for negative amplifier sense (−) input, while stimulating with the same settings as the benchtop tests from E3 to case. By doubling the surface area of one of the sensing electrodes this should theoretically double the capacitance mismatch between the two sensing channels.

##### (d) Classifier characterization

To validate the effect of the slow transient responses of the different capacitor mismatches on the fast aDBS mode in the DyNeuMo-2. An optimized version of the fast adaptive algorithm first introduced by [20] was implemented in DyNeuMo-2. The DyNeuMo-2 digital signal processing chain contains a high pass filter set to remove the DC offset and slow transient response signals, and then the data is fed through a 4th order bandpass filter set to detect a Beta signal at 20 Hz with a cut off frequency of 18–22 Hz. Then the data is rectified and smoothed to detect the power within the frequency band of interest (supplementary material figure S8).

#### 1.3.2. Simulation results

The DyNeuMo-2 and the RC + S sensing circuits with tissue electrode interface capacitor mismatches of 680 nF–1.47 *μ*F and 1–2.2 *μ*F (supplementary material, table S3 tests 2,3) showed a similar slow transient response profile during stimulation switching ON–OFF. The values of the selected capacitors resulted in a settling time of about 1000–1200 ms for both sensing circuits, with differences in the maximum amplitude of the signal (about two times larger in DyNeuMo than in RC + S) (supplementary material, figure S9). Moreover, the transient response of capacitor values 680 nF–1.47 *μ*F and 1–2.2 *μ*F, is very similar to the majority of transient responses observed in patient recordings with the RC + S (figure 8). The test with capacitor values of 150–330 nF (supplementary material, table S3 tests 1) resulted in a different transient response to what was observed in patient recordings, with a much faster settling time of 200–300 ms. This suggests that these capacitor values are unlikely to be encountered in the DBS tissue–electrode interface *in vivo*.

#### 1.3.3. Benchtop RC-R interface and saline test results

**Figure 10.**
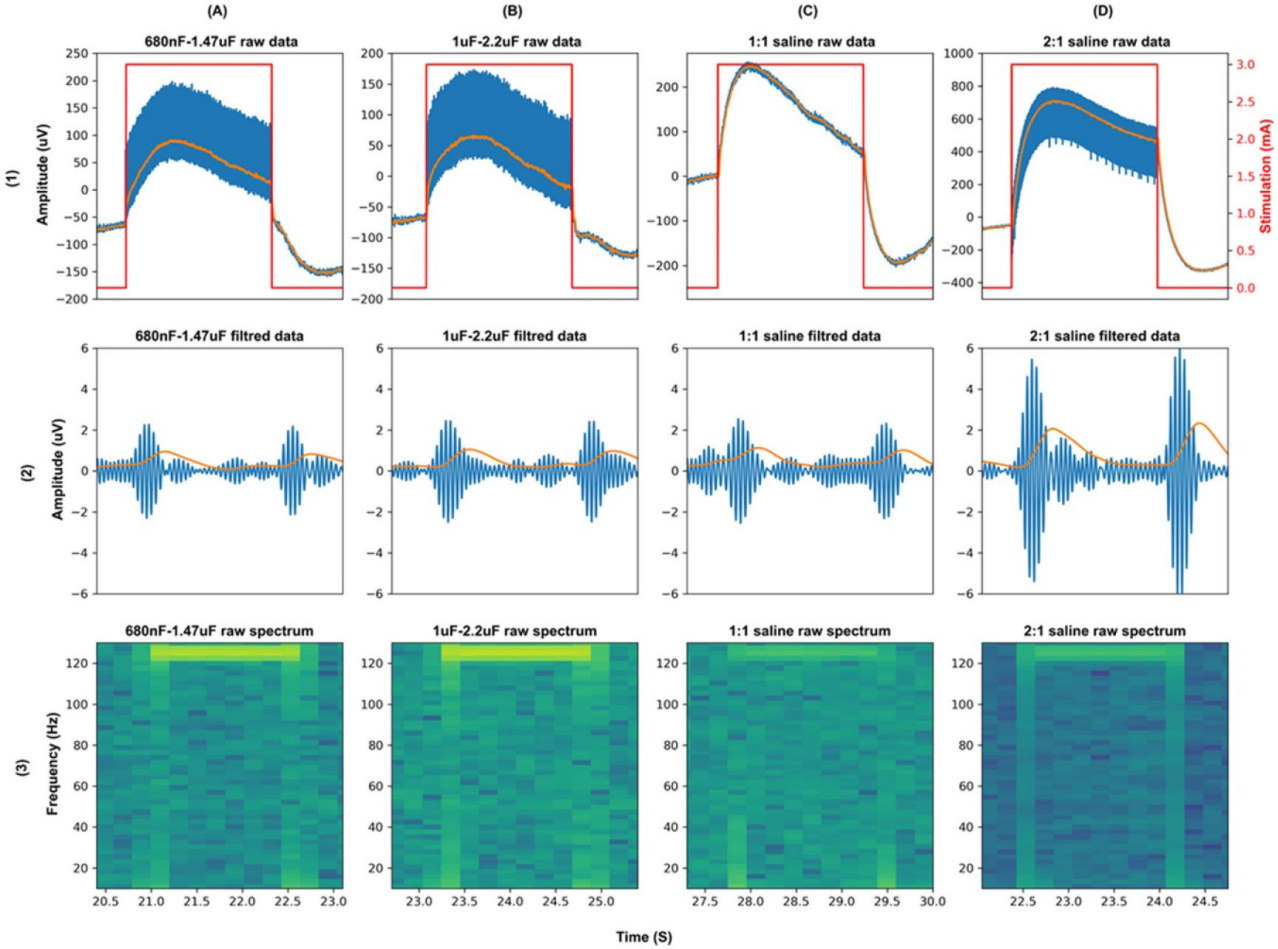
DyNeuMo-2 benchtop and saline test results. (A) Transient response results of RC-R network with 680 nF–1.47 *μ*F capacitor mismatch. (B) Transient response results of RC-R network with 1–2.2 *μ*F capacitor mismatch. (C) Saline test transient response with 1:1 electrode no surface area mismatch. (D) Saline test transient response with 2:1 electrode surface area mismatch.

The transient response of the DyNeuMo-2 IPG for the different pairs of tissue electrode interface capacitance is illustrated in (figure 10). A stimulation ON–OFF transient response with a settling time between 700 and 1000 ms was measured (figures 10(A)-(D)). This is similar to the results observed in the Spice simulation with slightly faster settling time periods. The benchtop and saline tests transient responses are similar to the patient data recorded with the RC + S illustrated in figure 8. Furthermore, from the spectrogram of the raw data illustrated in figure 10(row 3), we see that the slow transient response results in an increase in power across the frequency range when stimulation switches ON–OFF. This increase in power only lasts for about 250–350 ms. The output from the DyNeuMo-2 power band classifier for the different impedance mismatches is illustrated in figure 10(row 2). The stimulation ON–OFF transients causes the bandpass filter stage to ring in all cases (similar to the DC transients observed in data from RC + S subjects). The ringing of the bandpass filter lasts for approximately 250–350 ms, which is the settling time of the 4th order Butterworth filter. The envelope power detection signal shown in orange in row 2, settles down to the baseline noise within 1000 ms from the stimulation ON–OFF, this is due to the 400 ms moving average smoothing filter, which is implemented as a 1 Hz 2nd order lowpass filter. The results also demonstrate that even in a homogeneous saline solution there is a small capacitance mismatch in the saline-electrode interface, which results in a slow transient response. Moreover, the different capacitor mismatch values affect the maximum amplitude of the ringing in filtered data more than the duration (similar to RC + S subjects, figure 8). The benchtop and simulation test results identified the limitation of the sensing channels of implanted aDBS devices during stimulation ON–OFF transitions, and how it would affect the operation of a fast aDBS algorithm. Identifying and characterizing these limitations would inform the hardware and software tuning required in implanted aDBS devices to perform fast aDBS algorithms.

### 1.4. Checklist validation: ‘fast’ aDBS implementation with RC + S (*in vivo*)

After verification of the sources and mitigations of the stimulation transient, we were able to implement ‘fast’ aDBS in which stimulation was successfully adjusted based on underlying physiology rather than ramp artifact. In a PD-STN patient (72 year-old man, 8 years history of PD), we studied if trade-offs in the analog chain and the time/frequency classifier could reduce stimulation transients and mitigate its effect in algorithm performance (e.g. reducing false detections or reentrant loops). We did so by applying the checklist defined in figure 3 and table 1 (note the setting values in the bullet list below are tailored to the example described herein):

- Analog Sense. High-pass filter corner of 8.6 Hz instead of 0.85 Hz, reducing the duration of the transient response due to stimulation transitions yet ensuring sufficient spectral bandwidth to detect beta band power.
- Digital Signal Processing. Reducing FFT time window (64 points, 256 ms) and interval (50 ms), still keeping enough frequency resolution (~4 Hz). Note this is similar to time/frequency trade/offs derived from the simulation filter/classifier analysis with the DyNeuMo (see figures 4 and 12).
- aDBS Detector/Classifier. Choosing detector blanking (550 ms) with sufficient duration to avoid reentrant loop due to transient during stimulation ramp up/down (250 ms), while not blanking so long so as to miss the next burst (in STN-PD next bursts may appear within ~500 ms). Other detector/classifier settings: 100 ms update rate (average of two consecutive power band values, i.e. increasing signal to noise ratio (SNR) yet keeping overall detection duration at ~500 ms).
- Stimulation modulation. Minimizing the stimulation transition range (2→2.6 mA) to reduce magnitude of transient during a 250 ms stimulation ramp duration. We started at a nonzero amplitude level to avoid under-stimulation and increased amplitude to the maximum level tolerable by the patient. As shown in previous sections (e.g. figure 8, supplementary material figure S3), a lower range of stimulation transitions leads to reduced stimulation transients.

**Figure 11.**
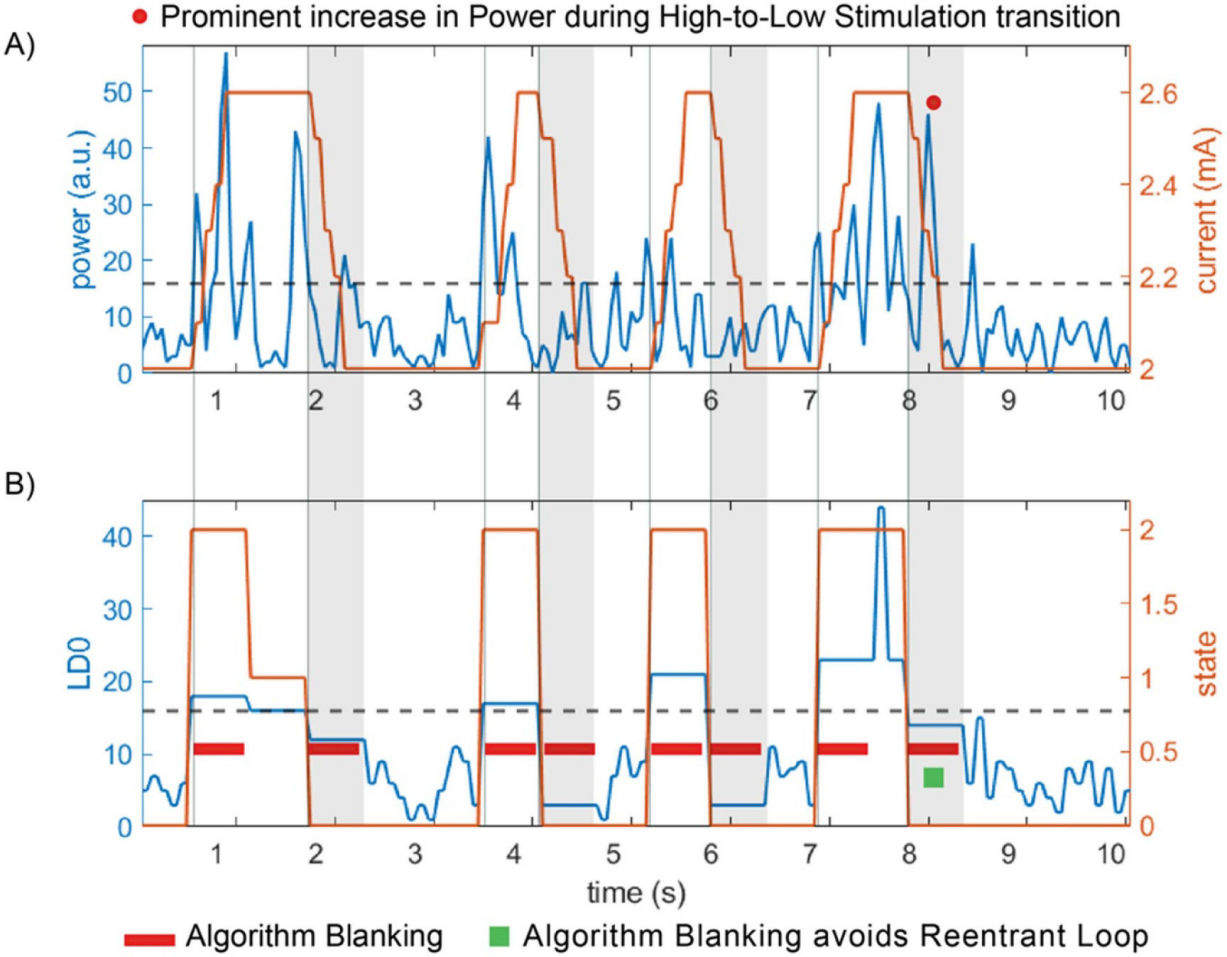
Implementation of ‘fast’ aDBS in a chronically implanted patient with RC + S using optimal settings. (A) Input power feature to detector (blue trace) and corresponding transitions of stimulation current (orange trace) following power threshold crossing (dashed black line). (B) The linear discriminant LD0 (blue trace) is the averaged output of the power (blue trace is the average of 2 FFT power values in (A)). The algorithm blanking segment (lockout period) of 550 ms at the onset of a threshold cross (states 0–2 and 2–0) is depicted with a red rectangle. The detection state (orange trace) changes value with threshold crosses of LD0, with state 2 indicating a detector increase, state 1 (‘hold’) indicating LD0 within thresholds (in case upper and lower threshold) and state 0 indicating a detector decrease below threshold event. Note: after second 8, the sudden stimulation transition from high to low creates a prominent increase in power, with algorithm blanking of 550 ms or shorter (e.g. 250 ms, duration of the ramp) sufficing to avoid reentrant loop.

A representative 10 s segment of the ‘fast’ aDBS detector using the settings above is shown in figure 11. The appearance of the stimulation ramp or transitions does not result in reentrant loops. The raw LFP signal did not show large DC transients during stimulation transitions (data not shown here), this was likely because of the combination of reduced stimulation range and use of a higher corner of the high pass filter (HPF). The appearance of detector state changes and consequently the delivery of adaptive stimulation and its duration varied with time (figure 11), suggesting that stimulation is triggered by physiological changes and not driven by the transient artifact after switching.

## 2. Discussion

Here we evaluate technical challenges in the implementation of chronic aDBS paradigms in an embedded (fully implanted) configuration. We focus on continuous (real-time) sensing during stimulation of the disease-or-symptom specific neural biomarker (full duplex configuration), in particular, technically demanding ‘burst trimming’ algorithms that operate on fast time scales (e.g.<1 s; trimming bursts as they occur). Fast algorithms are particularly sensitive to false detections due to the susceptibility for reentrant loops and limit cycles. We argue this is due to the interaction of the stimulation waveform with the DBS electrode-tissue interface and the step response properties of a high-pass filter at the front-end of the sensing IPG analog chain. To illustrate this phenomenon, we used patient data recorded with one of the most sophisticated aDBS devices, Summit RC + S (Medtronic), available only under investigator-initiated research protocols. We also developed an electrical model that mimics ‘real world’ considerations of the patient-electrode-tissue interface and analog high pass filter characteristics.

### 2.1. Active versus passive recharge

A stimulation pulse can be applied with an active (active recharge) or a passive (passive recharge) anodic phase. In both ‘active recharge’ and ‘passive recharge’, the cathodic stimulation phase returns to zero reference immediately after pulse width duration [49]. Passive recharge pulsed stimulation provides an exponential decay from anodic peak amplitude to zero reference. A passive recharge stimulation scenario is beneficial to reduce battery power consumption, as no active electronics are required to drive the recovery. Passive recharge is often the only mode of operation available on primary cell neurostimulators such as Activa PC + S or Percept PC. While stimulation is being delivered, stimulation and sensing engines are referenced to the same electrical ground, the implant case. The net result is a longer time segment where both the stimulation artifact is recovering on the recharge pulse which must be rejected by the pre-amplifier, and increased exposure to physiological artifacts that can couple through the IPG such as the ECG signal.

### 2.2. Cardiac artifact

In addition to stimulation artifacts, cardiac artifacts may preclude application of adaptive stimulation algorithms by corrupting signal spectral information in the low frequency biomarker bands [50, 51]. The presence of an ECG artifact is only observed in the subcortical channels, and is not present in cortical channels. This may be due to a number of factors, for example signal-to-noise ratio, where cortical signals are ~10 larger than subcortical signals helping to obscure the ECG/Stim artifacts, or use of different input bores on the RC + S (i.e. depth channels are placed in Bore 0, and cortical channels are placed in Bore 1, physically separating the stimulating electrode wire from the cortical recording wire). The most likely explanation is the relative size of the signal, as the common mode rejection can be compromised enough to allow ECG feedthrough of cortical signals, such as in the extreme case where the IPG case is one input of the differential chain [52]. The use of active recharge can reduce the magnitude of the ECG artifact. However, active recharge requires higher power to deliver the same amount of stimulation energy and thus is most applicable to rechargeable pulse generators. Another mitigation to reduce ECG artifacts is to place the sensing pulse generator further away from the heart. Neumann *et al* [50] present results of a multicenter study demonstrating considerable reduction of ECG artifact with devices placed on the right chest of patients. Other studies have opted for pulse generators placed farther away from the chest, for example skull based implants [1, 5, 7, 30]. A mitigation strategy during ‘off-line’ (post processing) has been proposed by [51] using template-based extraction, but this implementation is not applicable to closed-loop (real-time) DBS.

### 2.3. Sense blanking

The high frequency stimulation artifact can be managed by increasing the sense-blanking value while simultaneously using active recharge; the largest amplitude artifact from stimulation for the Summit RC + S occurs using passive recharge with a 0.335 ms blanking value (the shortest possible value). In contrast, the DC transient artifact during ramping is relatively unaffected by any of the combination of settings used, suggesting this is related to overall redistribution of charge from changes in stimulation state versus a single pulse-related source. The net effect of the stimulation transient is a broadband power increase during stimulation changes, which may interfere with power-based discriminators. Note that for the Summit RC + S and Activa PC + S, the high-pass filter corner (0.85, 1.2, 3.3 or 8.6 Hz) is on the same order as ramp-transitions for stimulation with time step increments from 100 ms (fastest) to 1000 ms (slowest). To minimize extended artifact effects, the high-pass filter corner should be set to as high a corner as possible without compromising the physiological biomarker of interest, or the input impedance of the sensing chain. For a fixed input coupling capacitor, a higher filter corner lowers the input resistance proportionately. To avoid this impact, an additional filter might also be implemented in the digital processing chain to avoid compromising the front-end impedance characteristics.

### 2.4. Transient response: avoiding limit cycles with algorithm blanking

The transient response observed in patients had a shorter duration with increased ramp rate (mA s^−1^) until reaching a minimum steady level (from ~1.0 up to 4 mA s^−1^) when comparable amplitude ranges were evaluated (figure 8). While the magnitude of the transient artifact can be reduced by reducing difference between low and high stimulation amplitude limits, the overall duration is dominated by the time constant of the capacitive elements of the input analog sensing circuitry. To reduce the time constant of the transient response in the RC + S, the high-pass filter corner can be adjusted depending on the biomarker band of interest (HP corners: 0.85, 1.2, 3.3 and 8.6 Hz). Note that the degree of impedance mismatch influenced the magnitude of the transient step response (larger mismatch, larger amplitude of the DC transient) but not the duration (see figure 8 similar ramp rates (e.g. (a)-(c)) and figure 10 middle panels). This is likely due to the time properties of the analog high-pass filter at the input of the amplifier chain dominating over a shorter time constant of the tissue electrode impedance mismatch. In the RC + S, ‘algorithm or detector blanking’, can be applied to mitigate the effect of the transients during stimulation amplitude adjustments. In general, a blanking duration higher than the duration of the ramp is required to let the sense amplifier signal settle back to baseline. In ‘slow’ aDBS algorithms with biomarker state changes on the order of minutes to hours (e.g. medication fluctuations in PD or onset of seizures in epilepsy), mitigation of the transient response is possible using blanking periods of the duration of the ramp [6, 37, 53].

### 2.5. ‘Fast’ aDBS—considering classifier time-frequency trade-offs

The potential clinical benefit of fast aDBS was first demonstrated in brief perioperative studies with externalized DBS leads [20, 24]. In those proof of concept (perioperative) studies with externalized leads, an external bioamplifier (TMSI Porti 7) was used without analog input high-pass filters or additional input sense channel safety capacitors, thus a minimal transient step response during stimulation transitions. This type of external instrumentation comes with a higher dynamic range and does not require analog input high pass filters to ensure a trade-off between signal resolution and dynamic range. The ‘fast’ adaptive stimulation algorithm to shorten beta bursts in PD has not yet been clinically assessed in a fully embedded configuration due to the observed reentrant loop during concurrent stimulation and sensing. The risk of limit cycles is critical to consider when attempting to implement faster aDBS. To mitigate the technical implication of artifact-driven limit cycles during aDBS, attention needs to be paid to trade-offs in the analog chain (e.g. increase HP filter corner to reduce duration of transient), time/frequency resolution of classifier and careful choice of algorithm blanking duration. For example, by reducing the length of the FFT buffer, the impact of a transient response that could obscure detection of beta activity may be reduced with a reasonable detriment of frequency resolution. Finally, choosing an optimal value of algorithm blanking is key, it should be long enough to avoid reentrant loop but not too long to avoid missing the next pathophysiologic burst (e.g. 550 ms for the beta algorithm in figure 11). Here, we utilized these methods to demonstrate technically successful ‘fast’ aDBS in a patient with PD, without reentrant loops.

It should be noted that these observations are guided in part by the choice of signal chain architecture [54], and alternative methods for artifact suppression are under investigation. For example, the designer can use a ‘weak’ auxiliary stimulation signal sensed by the recording system [55] or apply an irregular sampling rate [56].

### 2.6. Considerations for next generation aDBS devices

#### 2.6.1. Optimizing management of the tissue–electrode interface

The observed stimulation ON–OFF transient response is a result of the high-pass filter in the front-end sensing circuit for the DyNeuMo-2 and RC + S. The use of a passive high pass filter on precision instrumentation amplifiers is not recommended as it degrades the common mode rejection ratio of the amplifier and reduces the input impedance (two main features and advantages of using instrumentation amplifiers). However, in implanted IPG systems the use of a passive high-pass filter is enforced as part of the single fault safety design for the stimulation channels. As IPGs include a large DC decoupling capacitor for the stimulation electrodes to ensure net zero DC current flowing through the DBS electrodes, using a passive high-pass filter in the front-end results in reducing the total input impedance to the value of the high-pass filter resistor. This also reduces the common mode rejection ratio of the sensing circuit. Furthermore, as illustrated in the simulation results supplementary in figure S9, the most dominant factor in the tissue–electrode interface impedance is the capacitor. The tissue–electrode interface capacitance is shown to be in the order of a few microfarads [45, 47, 48]. A mismatch in the capacitive element of the tissue electrode interface will result in a slightly different high-pass filter cut off frequency set on the positive and negative terminals of the sensing channels. This would result in each channel having a slightly different settling time on each terminal in the event of a step response i.e. stimulation switching ON–OFF. Hence, this would result in a slow transient response signal showing as a differential input to the amplifier. This issue can be mitigated by, (a) increasing the high pass filter resistor value to increase the input impedance, (b) reducing the value of the high pass capacitor to reduce the loading from the tissue electrode interface, as illustrated in supplementary figure S10.

#### 2.6.2. Time-frequency trade-offs in aDBS

**Figure 12.**
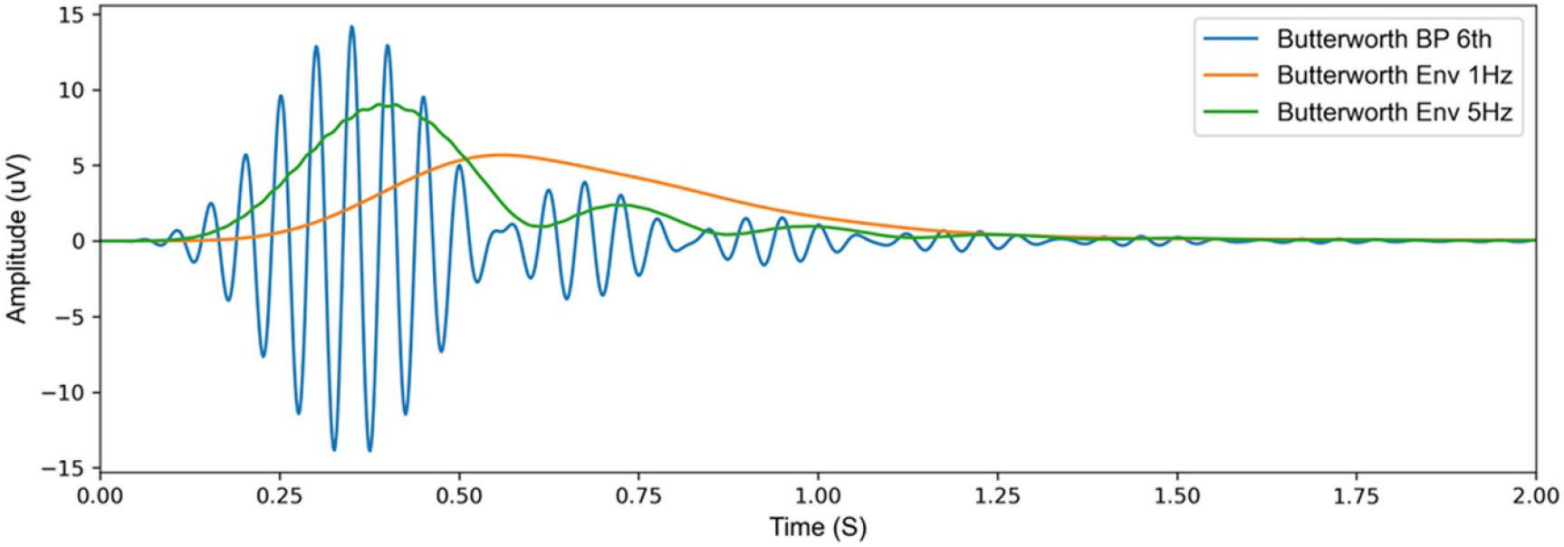
1 mV impulse response of a 6th order Butterworth with 1 and 5 Hz envelope detection filter.

An increase in ringing duration during digital signal filtering before the classifier could cause the algorithm to have a higher rate of false triggers (false detections due to reentrant loop effect). For this reason, the beta filter used in the DyNeuMo-2 is set as Butterworth 4th order with a bandwidth of 18–22 Hz (figure 4), which results in a reasonable frequency specificity and a reduced time domain ringing duration. Also, the power envelope smoothing filter needs to be designed carefully, as a slow smoothing filter designed to respond to beta bursts of 250–350 ms, will filter out shorter bursts and noise, which will prevent false triggers. Meanwhile, a burst of 250–350 ms such as the ringing caused by the stimulation ON–OFF transient response will result in the smoothing filter staying above baseline noise for about 1000 ms as illustrated in figure 12. This in turn will increase the required blanking period and limit how fast the aDBS algorithm can perform. However, this can be mitigated by reducing the smoothing filter window, with the compromise that this might lead to the classifier responding to shorter bursts of the signal of interest as illustrated in figure 12. Similarly, a shorter FFT window size in the RC + S detector combined with detector power averaging and optimal blanking could lead to a trade-off in frequency and time resolution, targeting burst activity and avoiding reentrant loop without missing subsequent pathophysiologic bursts (figure 11).

Although we have focused on applications based on DBS, ECoG based prosthetic systems would potentially benefit from the techniques described, especially those incorporating sensory feedback which might provide artifacts in the proximity of the sensing electrode. For example, a memory prosthesis described by Kahana [57-59] and closed-loop spinal cord stimulation to treat chronic pain [60] also rely on state-dependent, field-potential based algorithms and stimulation, where the bi-directional elements are required. Similarly for neuropsychiatry, a neuromodulation depression system might also benefit from a full-duplex approach [35, 36]. In summary, the methods and results described here are useful for a broader range of applications than classical DBS for movement disorders, where field potentials might be useful as the input. Ultimately, successful implementation of aDBS therapies in any application will require a deeper understanding of long-term, symptom-related biomarker dynamics [6, 61], as well as patient-specific control strategies that can be embedded in the implant device [23, 27, 62].

## 3. Conclusion

Artifacts during concurrent stimulation and sensing should be carefully studied in any applications of adaptive closed-loop DBS. When stimulating and sensing with electrodes in the same DBS lead during ‘fast’ (<1 s) aDBS, impedance mismatch of the sensing electrodes and filter properties of the analog chain need to be considered. Careful choice and optimization of analog and digital filter parameters in the detector engine and use of detector blanking strategies is then key to mitigate the effect of stimulation transients in the classifier performance. In closed-loop DBS applications with slower biomarker latencies, setting a detector blanking of at least the duration of the stimulation ramp may be sufficient to avoid algorithm reentrant loops during adaptive stimulation. In the future, the properties of the analog chain should include mechanisms to reduce the step transient responses during adaptive stimulation, and to to detect these reentrant loops and enable switching to constant (open loop) stimulation therapy to mitigate adverse clinical effects.

## Supplementary Material

jneac59a3supp1.pdf

## Figures and Tables

**Table 1.** Checklist for artifact mitigation of aDBS system with concurrent sensing and stimulation.

	Signal chain element	Mitigation strategy and potential trade-offs (vs)
1	IPG location to reduce ECG artifact	Right chest, cranial, use of active recharge stimulation vs power consumption
2	DBS electrode impedance	Minimize tissue electrode impedance mismatch, symmetric dipole arrangement
3	Frontend sensing	Leakage current, polarization management, use of active recharge stimulation, stimulation pulse ‘sense blanking’
4	Digital signal processing	Time vs frequency resolution trade-off in classifier, dynamic range management vs resolution floor
5	Adaptive DBS algorithm	Clock synchronization and ‘algorithm blanking’ (post-stim lockout period)
6	Stimulation modulation	Stimulation ramps and stimulation amplitude transitions vs response time and dynamic range of adaptive stimulation algorithm

## Data Availability

The data generated and/or analysed during the current study are not publicly available for legal/ethical reasons but are available from the corresponding author on reasonable request.

## Footnotes

11
https://github.com/openmind-consortium/App-aDBS-ResearchFacingApp
