# Supplementary material for: Concurrent stimulation and sensing in bi-directional brain interfaces: a multi-site translational experience
Source: J Neural Eng. Author manuscript; Available in PMC 2023 Mar 31. (PMC9095704; doi:10.1088/1741-2552/ac59a3)
Supplement: jneac59a3supp1.pdf [file NIHMS1794307-supplement-jneac59a3supp1_pdf.pdf]

Supplementary Material

1. Background

1.1. Susceptibility, how artifacts can limit performance

In an adaptive or closed-loop DBS system, stimulation is adjusted based on control feedback signals from neural biomarkers [1] or behavioral sensors [2]. In the scheme of aDBS based on concurrent neural stimulation and sensing, the correct modulation of stimulation is susceptible to artifactual coupling in the signal chain (main manuscript Figure 2 and Figure 3). Three main types of signal artifacts can degrade the neural (biomarker) signal in a chronic stimulation and sensing device [3]: a) concurrent stimulation [4–7], b) electrocardiogram (ECG or EKG) artifacts [8,9] and c) movement artifacts [10]. Any combination of these artifacts degrades or precludes sensing fidelity and detection of the biomarkers metric for detection algorithms. For example, during beta-based 'fast' adaptive stimulation in PD, algorithm performance can be challenged due to close proximity of the stimulation and sensing electrodes (subcortical DBS lead) and the relatively low signal levels of subthalamic beta bursts (1 to 20 microvolts). Most critical can be the coupling of transient step responses through the sensing channel during stimulation amplitude ramping (increments/decrements), with ranges of transient artifacts from 50 to 500 microvolts or higher (much higher than the field potential signal). Design choices within each block of the system can either help mitigate or exacerbate these artifacts.

1.2. General design considerations of the signal chain

The canonical signal chain provides a model for assessing sensitivity to artifacts. A key consideration of the management of sense-stimulation interactions within the tissue-electrode interface, which is the key point of coupling stimulation into the sensing chain. The electrodes are placed into neural substrates with variable impedance characteristics (Supplementary Material Figure S1(a)). The relationship of stimulation dipole fields to the differential sensing dipole is critical, and the tissue differences and electrode characteristics, resulting in impedance mismatch, can allow for the stimulation dipole to contaminate the measurement (Figure S1(b)). Choices made in the electronics interfaces -- both the stimulation waveforms, as well as sensing interface -- influence the degree to which artifacts impact sensing fidelity (Figure S1C).

Additional constraints beyond sensing algorithms further inform the system design. In designing a closed-loop adaptive stimulation device safety and power management are key aspects and should comply with regulatory requirements [6]. One key safety element is the stimulation source which carries the transfer of chemical energy from battery to the electrode-tissue interface in the form of a stimulation waveform [11]. To ensure no direct current (DC) charge is transferred to the tissue, which could harm electrodes and tissue, blocking DC capacitors are typically integrated in the input/output circuitry of the device (Figure S1(c)). In a multichannel stimulation and sensing intracranial device, this safety requirement applies also to

the sense channel, thus a blocking DC capacitor may also be part of the sense engine, which may also contain additional capacitor-resistor circuits serving as high pass filters. Interactions between stimulation waveform, mismatch of electrode-tissue impedances and filter characteristics of the amplifier chain will result in a transient (step) response (Figure 3(b)). These artifactual transient responses during variable stimulation and sensing will limit biomarker detection fidelity of a closed-loop system.

2. Benchtop Model and Setup

To facilitate a standardized, reproducible, and distributable lab testing environment, a benchtop electrode-tissue interface model was developed. The model was designed to optionally include any combination of the output impedance of the stimulator device, the return path impedance to the stimulator device case, and the electrode-tissue impedance for bipolar electrodes. All impedances were modeled as RC elements (an architecture published by [12–14]. The circuit was designed and simulated in LTSpice XVII (Analog Devices, Wilmington MA, USA). The modeled circuit is presented in Supplementary Materials Figure S5. In the initial stages of development, three use-cases were defined to the model: 1) to evaluate the performance of a closed-loop aDBS algorithm when physiologically relevant biosignals were provided, 2) to observe the response of closed-loop aDBS algorithms to physiologically relevant biosignals in the presence of an impedance mismatch, and 3) to “play back” recorded biosignals (where the electrode-tissue interface impedance effects were already present on the signal).

The model was designed to integrate as an attachment to the NeuroDAC [15], which handled the digital to analog conversion required to playback biosignals to an appropriate reproduction accuracy. Rather than simply providing one set of “balanced” and “mismatched” impedances respectively, the model was designed to facilitate on-the-fly switching of electrode and case impedances. A set of 7 impedance options for 8 circuit elements is provided, giving a total number of impedance combinations of 56 per bipolar electrode pair that can be rapidly selected without the need for soldering/desoldering. Input and output connections were selected to be stimulator agnostic. That is, the stimulator connections, and impedance outputs can connect to a great number of stimulating / sensing electrodes using simple BNC adapters. The benchtop PCB design is presented in Supplementary Materials Figure S5 (c).

3. Supplementary Tables

Table S1: Time requirements for allowable algorithm/detector blanking per aDBS use case given expected signal range (microvolts) and time ranges of disease specific biomarkers. In the first row typical target locations for each use case are listed representing how diverse and ubiquitous DBS has become.

	**PD**	**ET**	**Dyst**	**Epi**	**CP**	**OCD**	**Dep**
Targets (all subcortical)	STN, GP	VIM	STN, GP	CMT, ANT, STN, HIP, CB	PVG/PAG, VPL/VPM, IC	VC/VS, VALIC	VS/NAc, SCC, ITP, RCC, LHB
Typ. signal range at target location	1-50 uV	1-20 uV	1-50 uV	1-100 uV*	1-20 uV	1-20 uV	1-20 uV
Time scales of disease-specific biomarkers	<0.4s**<br><30s***<br>~hours****	0.5-2.5s	<0.4s**<br><30s***<br>~hours****	>1s	2-5s	>1s	>1s
Allowable detector blanking time	<0.5s (‘fast’)<br><30s (‘slow’)	0.5-2.5s	<0.5s (‘fast’)<br><30s (‘slow’)	>1s	2-5s	>1s	>1s

PD: Parkinson's disease, ET: Essential tremor, Dys: Dystonia, Epi: Epilepsy, CP: Chronic pain, OCD: Obsessive Compulsive Disorder, Dep: Depression, STN: subthalamic nucleus, GP: globus pallidus, VIM: ventral intermediate nucleus of the thalamus, CMT: centromedian nucleus of thalamus, ANT: anterior nucleus of thalamus, HIP: hippocampus, CB: cerebellum, PVG/PAG: Periventricular grey/Periaqueductal grey, VPL/VPM: ventral posterior lateral / ventral posterior medial nuclei, IC: Internal capsule, VC: Ventral capsule, VS: Ventral striatum, VALIC: ventral anterior limb of the internal capsule, NAc: Nucleus accumbens, SCC: Subgenual cingulate cortex, ITP: inferior thalamic peduncle, RCC: rostral cingulate cortex, LHB: lateral habenula.

* Epileptic seizure spikes

** pathophysiology bursts

*** behavioral

**** medication cycles

Table S2: Subject data demographics and datasets presented in this work.

Subject ID (paper)	Subject ID (site)	Disease	DBS Target	Study Site	Registration Number	Institutional Review Board (*)	Implantation date (M.Y)	Datasets presented in this work			
								Impedance	ECG artifact with Stim ON	Transient response	
										During Stim Ramping (Distributed)	During Fast aDBS (Embedded)
1	RCS_ET01	ET	VIM	UF	NCT02649166	201501021	May.20	✓	✓	✓	
2	RCS_ET02	ET	VIM	UF	NCT02649166	201501021	Jun.20	✓		✓	
3	RCS_ET03	ET	VIM	UF	NCT02649166	201501021	Sep.20	✓		✓	
4	RCS01	PD	STN	SU	NCT04043403	52548	Mar.20			✓	
5	RCS02	PD	STN	UCSF	NCT03582891	18-24454	Apr.19	✓			
6	RCS03	PD	GP	UCSF	NCT03582891	18-24454	Jun.19/Jan.20	✓			
7	RCS04	Dyst	STN	UCSF	NCT03582891	18-24454	Jul.20	✓			
8	RCS05	PD	STN	UCSF	NCT03582891	18-24454	Jul.19	✓			✓
9	RCS06	PD	STN	UCSF	NCT03582891	18-24454	Oct.19	✓			
10	RCS07	PD	STN	UCSF	NCT03582891	18-24454	Sep.19	✓			✓
11	RCS08	PD	STN	UCSF	NCT03582891	18-24454	Jan.20	✓			
12	RCS09	PD	GP	UCSF	NCT03582891	18-24454	Apr.20	✓			✓
13	RCS10	PD	GP	UCSF	NCT03582891	18-24454	May.20	✓			
14	RCS11	PD	STN	UCSF	NCT03582891	18-24454	Oct.20	✓			
15	RCS12	PD	STN	UCSF	NCT03582891	18-24454	Oct.20	✓			✓
16	RCS13	Dyst	GP	UCSF	NCT03582891	18-24454	Dec.21	✓			
17	RCS14	PD	GP	UCSF	NCT03582891	18-24454	Mar.21	✓			

UF: University of Florida; SU: Stanford University; UCSF: University of California San Francisco. VIM: thalamic ventralis intermedius nucleus; STN: subthalamic nucleus; GP: globus pallidus. *IRB under a physician sponsored investigational device exemption from the FDA (IDE #180097).

Table S3: Tissue electrode interface values used for DyNeuMo-2 bench top testing

	C1-C3	R1-R3	C2-C4-C5	R2-R4-R5	IPG
Test 1: capacitor mismatch 150nF-330nF	150nF – 330nF (±2%)	82kΩ (<±1%)	150nF (±2%)	82kΩ (<±1%)	DyNeuMo-2
Test 2: capacitor mismatch 680nF-1.47uF	680nF – 1.47uF (±2%)	82kΩ (<±1%)	680nF (±2%)	82kΩ (<±1%)	DyNeuMo-2
Test 2: capacitor mismatch 1uF-2.2uF	1uF – 2.2uF (±2%)	82kΩ (<±1%)	1uF (±2%)	82kΩ (<±1%)	DyNeuMo-2

Table S4: DyNeuMo-2 IPG stimulation configuration for Bench top and saline testing

Stimulation current	3mA
Stimulation mode	Monopolar E2 to Case Benchtop test<br>Monopolar E3 to Case Saline test
Stimulation pulses	100us biphasic
Stimulation frequency	125Hz
Stimulation period	1.6 seconds ON, 2.4 seconds OFF
Stimulation Ramping	No ramping (worst-case scenario)

4. Supplementary Figures

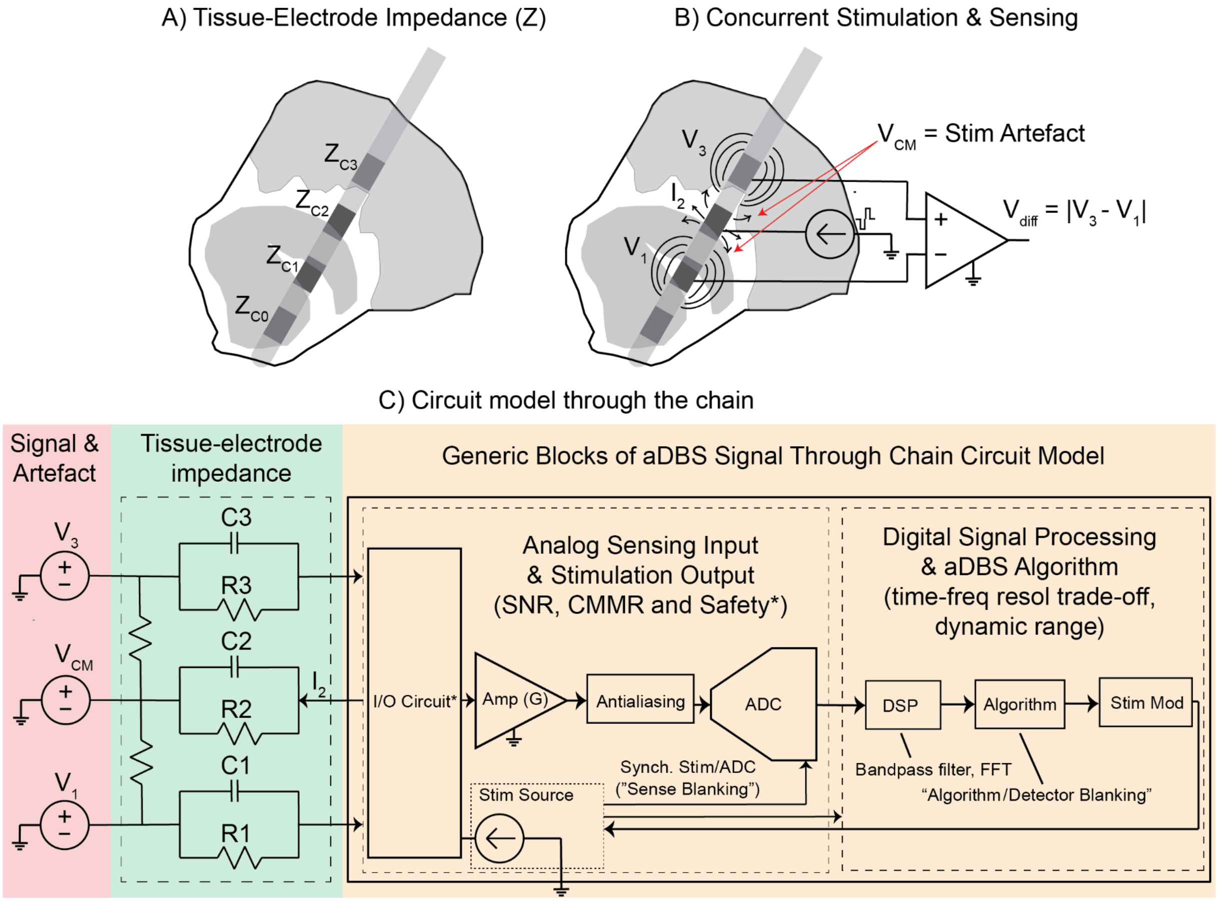

*needs to ensure safe/fail operation mode and minimal leackage currents within regulatory safety limits

Figure S1. Canonical signal chain providing a model for assessing sensitivity to artifacts. A) Electrodes are placed into neural substrates with variable impedance characteristics. This sketch represents a subcortical lead in the GP with contact C2 at the intersection of the external and internal structures GPe and GPi, B) relationship of stimulation dipole fields to the differential sensing dipole is critical, and the tissue differences and electrode characteristics, resulting in impedance mismatch, can allow for the stimulation dipole to contaminate the measurement (common mode artifact). C) The differential (local field potential) and common mode signal (in this example the stimulation artifact) interface with the sensing circuitry via the tissue-electrode impedance. Choices in the electronic interfaces - both the stimulation waveforms, as well as sensing interface - influence the degree to which artifacts impact sensing fidelity and how each independent block can be tuned for artifact mitigation.

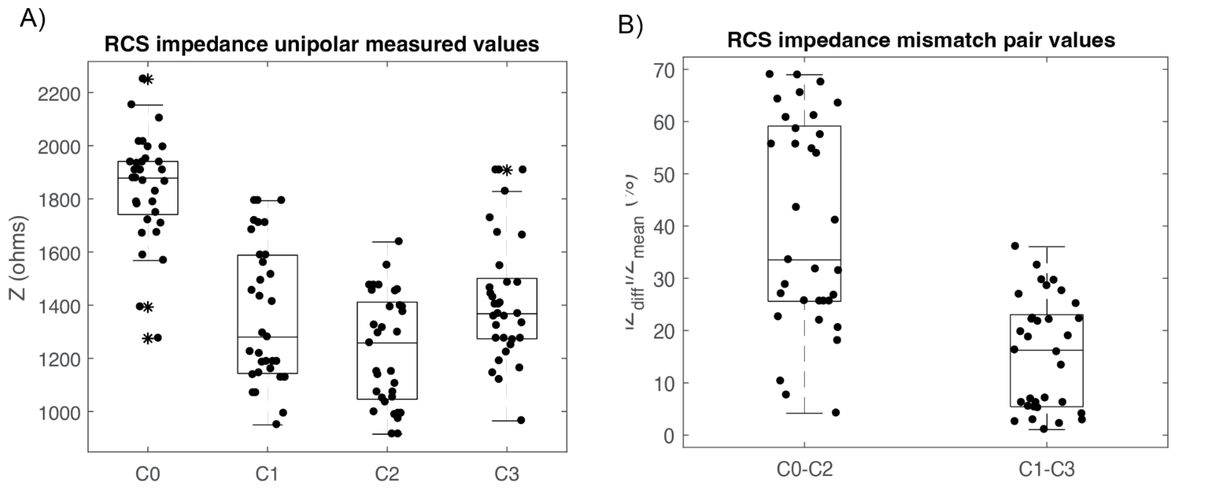

Figure S2. A) Distribution of single measurements ranging from 900 to 2250 ohms. Median impedance values are 1878, 1280, 1258, and 1368 ohms for contacts 0 through 3, respectively. B) Impedance mismatch values, same as B). Median calculated impedance mismatches were 33.53% (75th percentile 59.13%) for C0-C2, and 16.25% (75th percentile 23.04 %) for C1-C3.

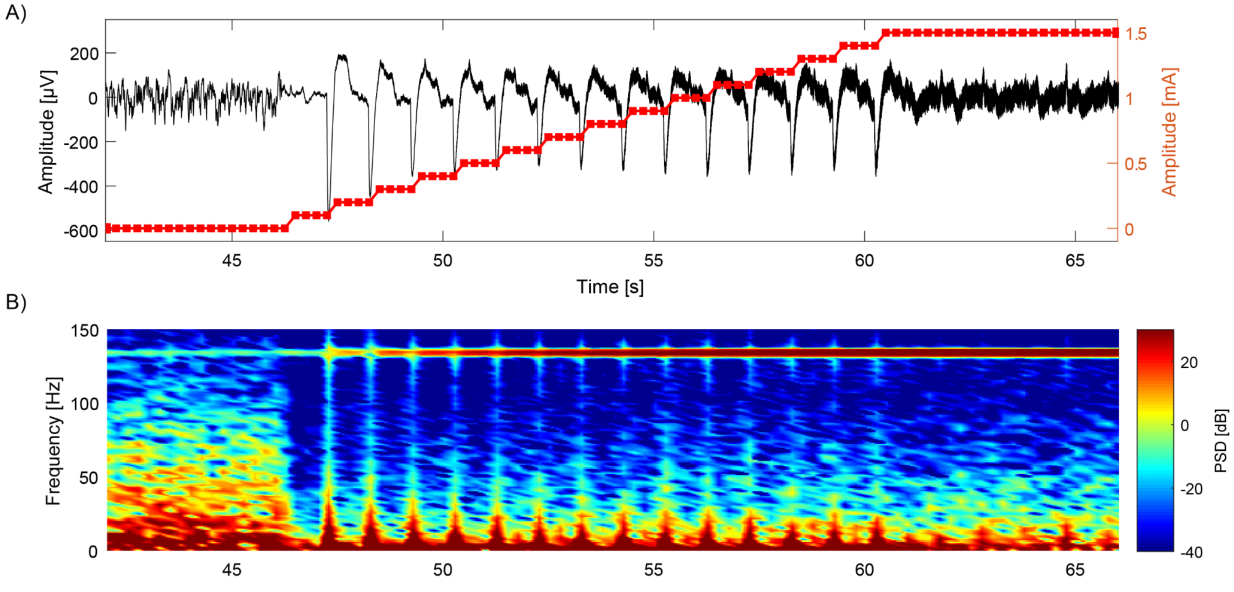

Figure S3A. Step response artifact on local field potential during 'slow' stim ramping at 0.1 mA/s. A) Monopolar stimulation from contact 2 (C2-/Case+), ramping from 0 to 1.5 mA over 15 seconds, stimulating at 135 Hz, 90 μs pulse width, and using active recharge. Recording from a bipolar sandwiched configuration (C3-C1) around the stimulating electrode, at 500 Hz sampling frequency. B) The spectrogram of the time-domain signal that is shown in A. Electrode placement is the same as in Figure 6.

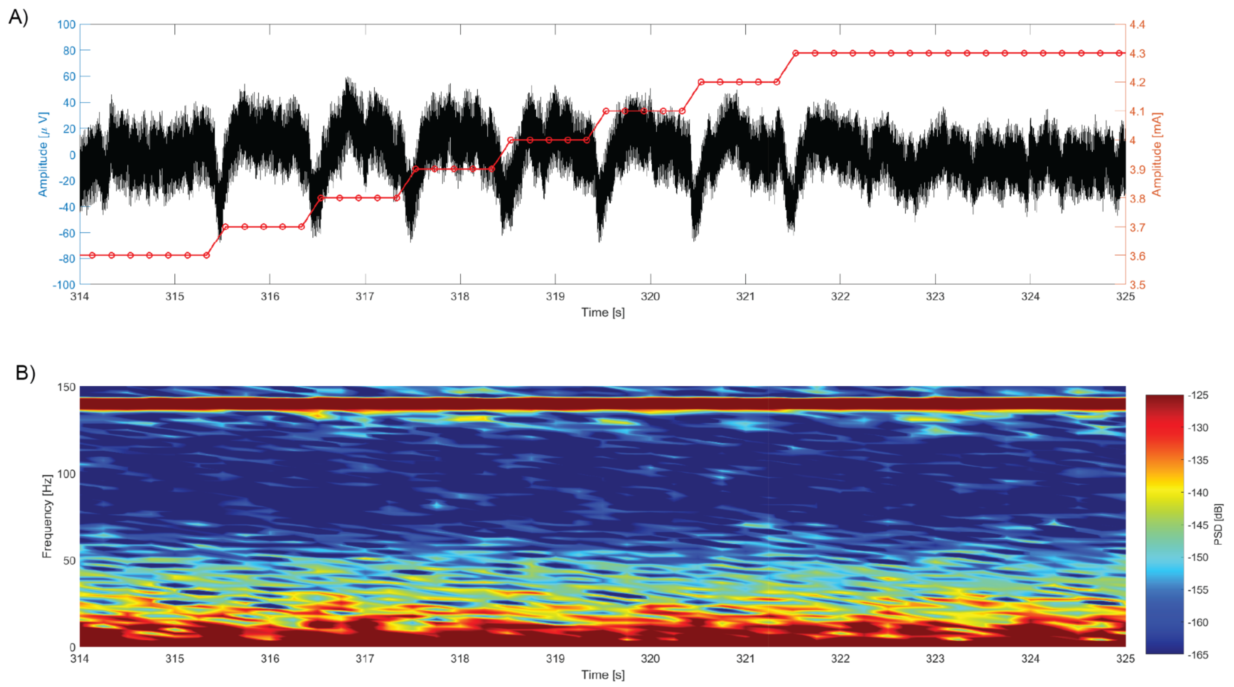

Figure S3B. Step response artifact on local field potential during 'slow' stim ramping at 0.1 mA/s when starting at a higher amplitude (3.6 mA instead of 0 mA). A) Monopolar stimulation from contact 1 (C1-/Case+), ramping between 3.6-4.3 mA over 8 seconds, stimulating at 140.1 Hz, 60 μs pulse width, and using active recharge. Recording from a bipolar sandwiched configuration (C0-C2) around the stimulation electrode at 500 Hz. B) The spectrogram of the time-domain signal that is shown in A.

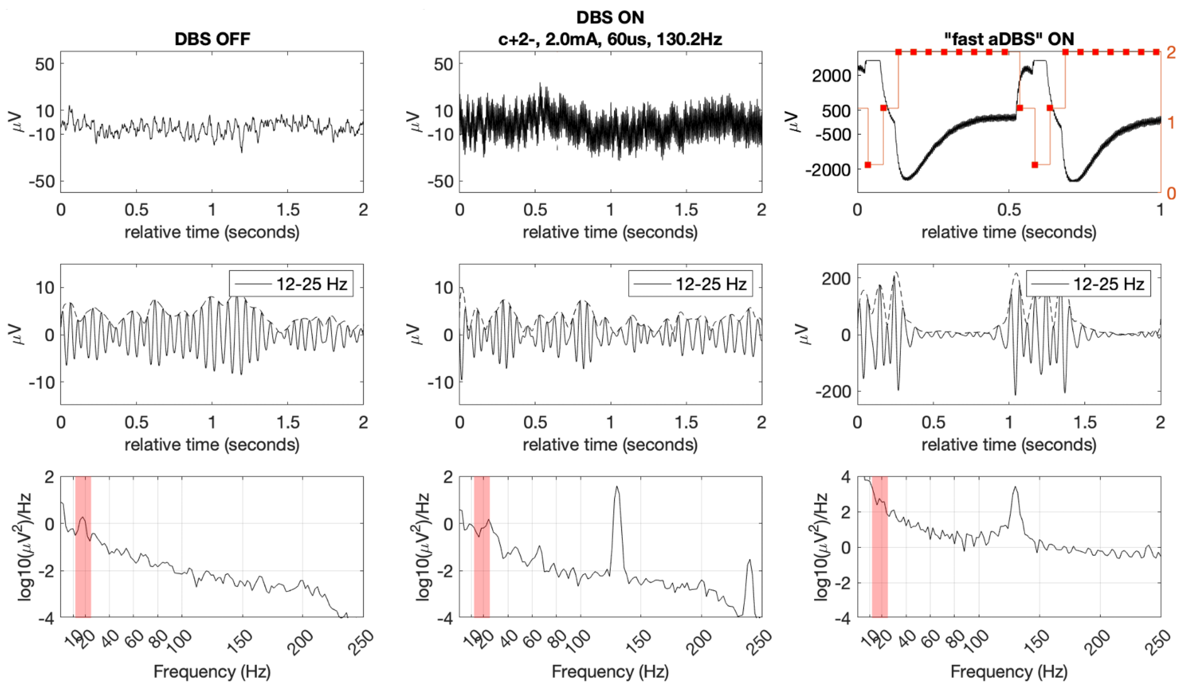

Figure S4A. Exploration of stimulation artifact on an STN patient (RCS05 right hemisphere). DBS OFF, DBS ON and ‘fast aDBS’, showing the extreme case of amplifier saturation during stimulation ramping.

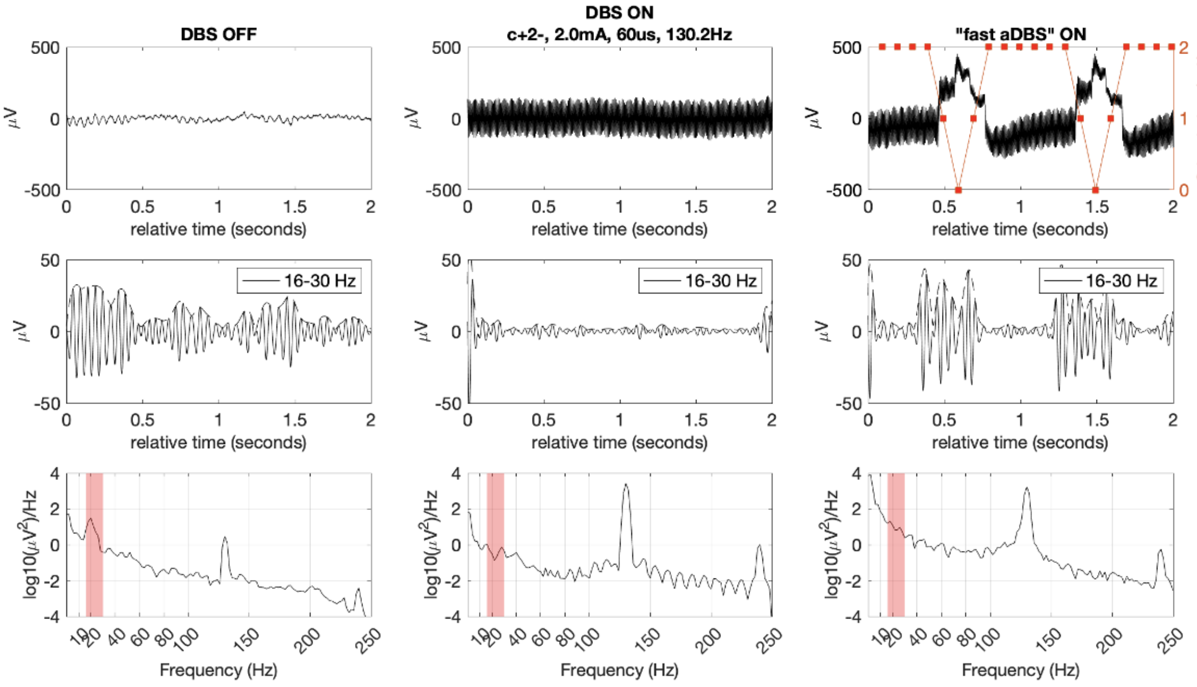

Figure S4B. Exploration of stimulation artifact on an STN patient (RCS07 right hemisphere). DBS OFF, DBS ON and ‘fast aDBS’, showing the limit-cycle transient on the raw and biomarker band-filtered data during stimulation ramping.

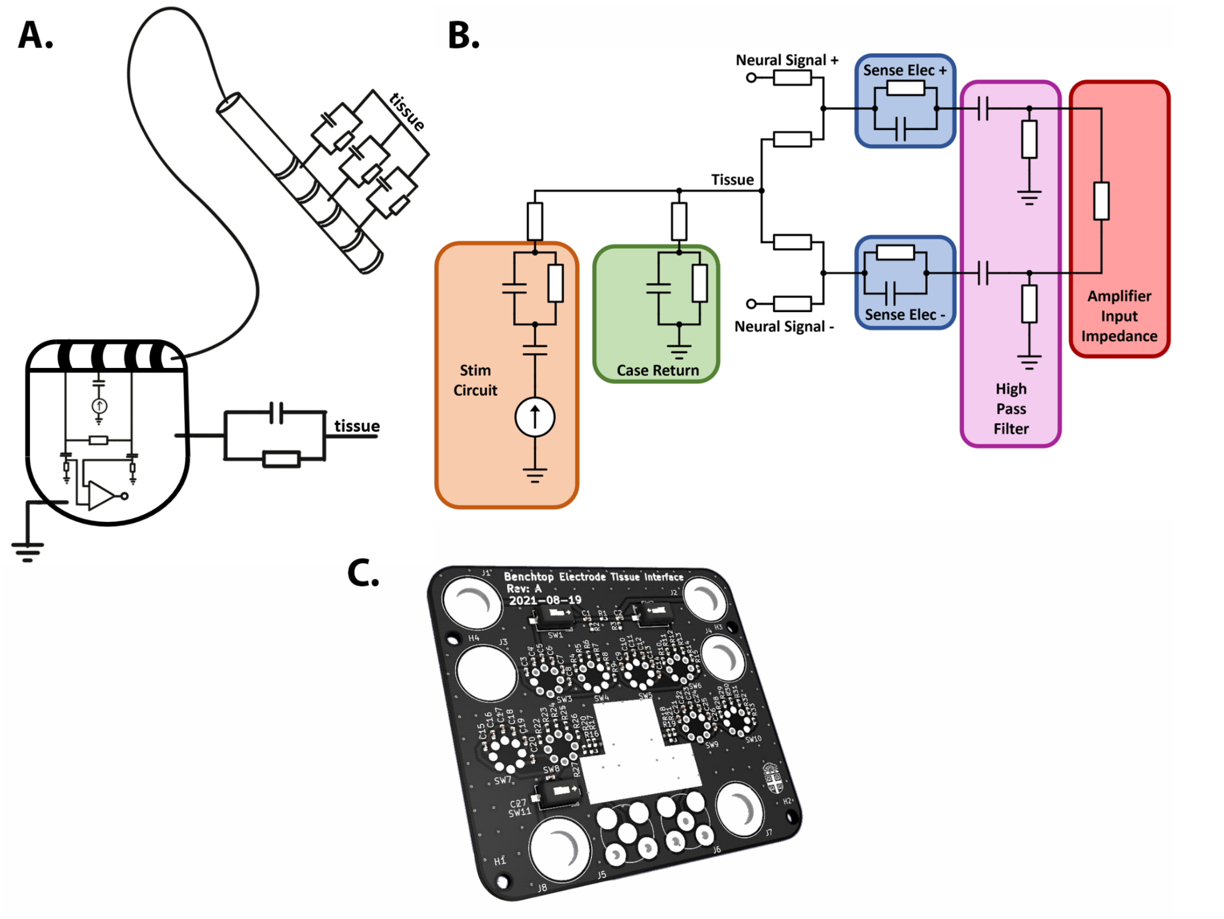

Figure S5. The simulated circuit of the standardized tissue-electrode impedance benchtop model. The tissue-electrode interface for all stimulation and sensing electrodes is modeled as an RC-R circuit. The values of resistors and capacitors are shown in Figure S6 and Table S3 (e.g., R = 82kΩ in parallel with C = 680nF-2.2uF and in series with R =1.5kΩ). The Stimulator case return impedances has the same conifugration with only difference in the series resistor R set to 100Ω. The stimulator current source is modeled by I1, and the NeuroDAC biosignal source is modeled by V1. Details of the design specification files are found on https://github.com/openmind-consortium/NeuroDACLoadPCB.

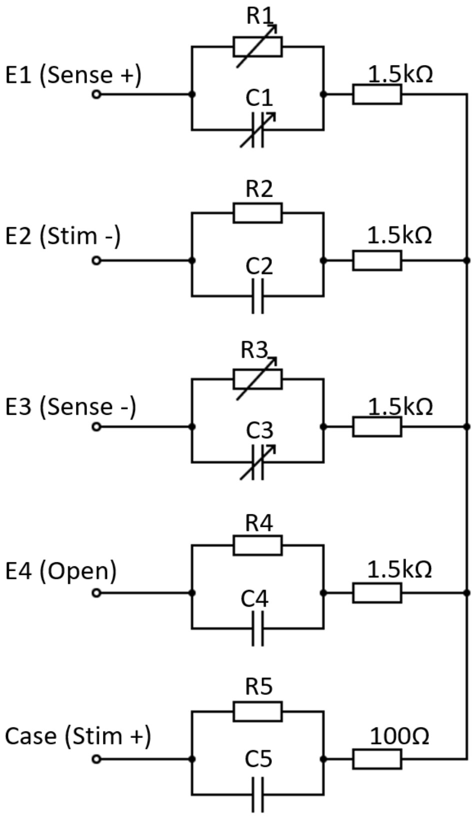

Figure S6. RC-R tissue-electrode interface star network setup for benchtop testing (see Table S3 for values).

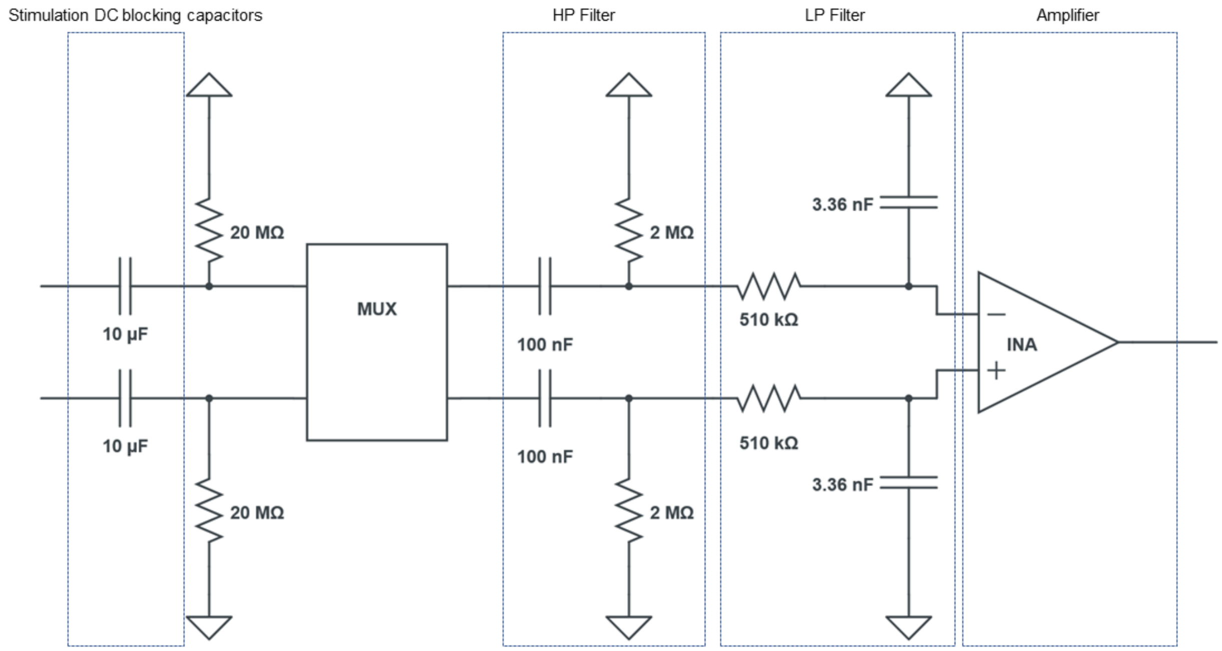

Figure S7. RC+S front end circuit (*reproduced with permission from the original authors* [6]).

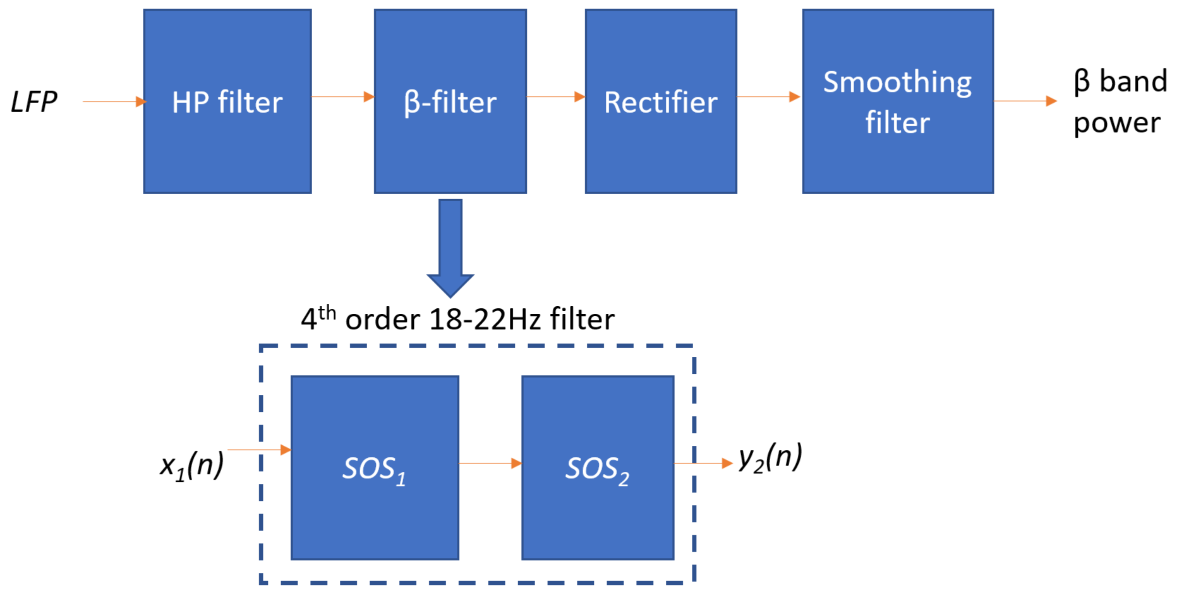

Figure S8. DyNeuMo-2 digital signal processing for Fast adaptive DBS.

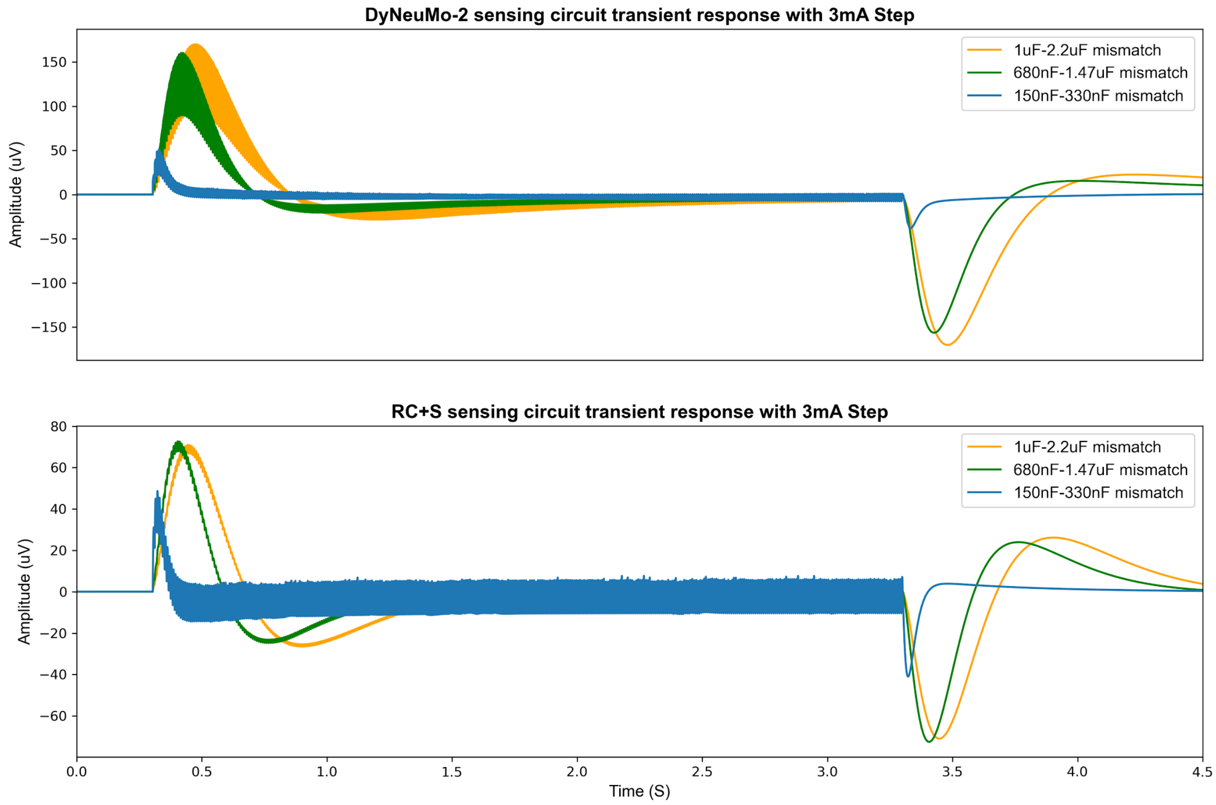

Figure S9. LTSpice simulation results of RC+S and DyNeuMo-2 front end sensing circuits with different tissue electrode capacitor values with 3mA, 125Hz, 90us biphasic stimulation without ramping.

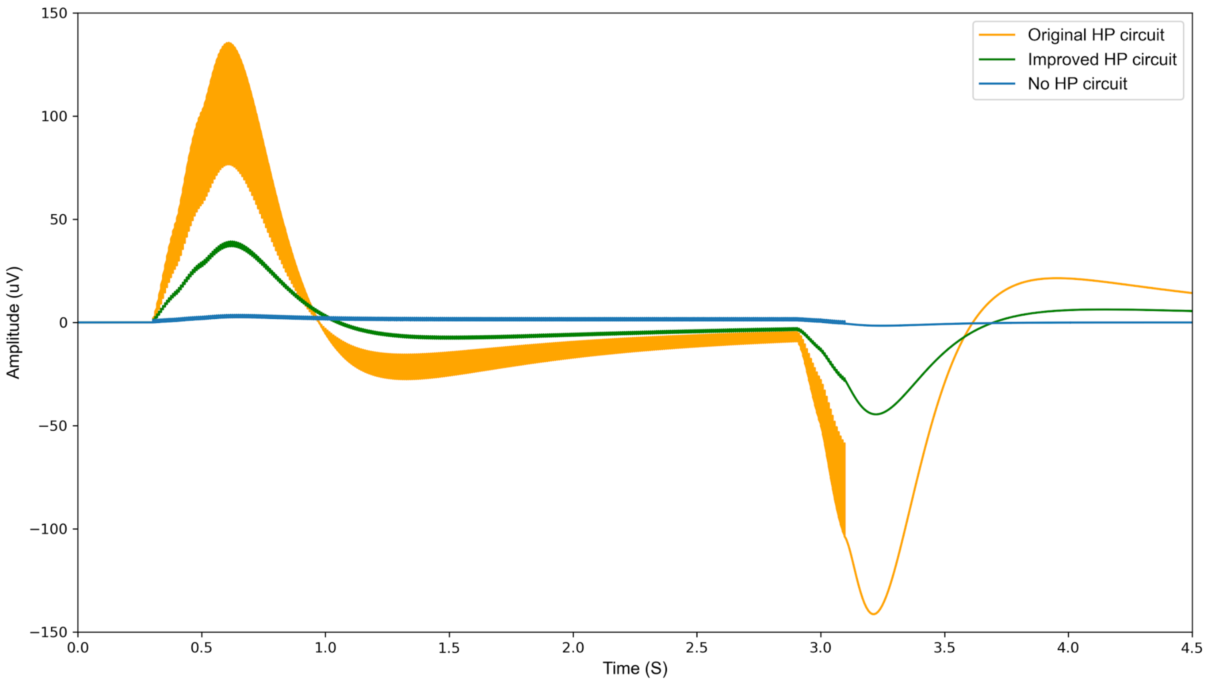

Figure S10. LTSpice simulation results of DyNeuMo-2 with different front end sensing circuits with capacitor mismatch of 120%. 1) DyNeuMo-2 original circuit (orange trace), 2) DyNeuMo-2 improved passive high pass filter (R=5MΩ, C=100nF) (green trace), 3) DyNeuMo-2 with no HP filter (blue trace).

5. Supplementary References

[1] Little S, Pogosyan A, Neal S, Zavala B, Zrinzo L, Hariz M, Foltynie T, Limousin P, Ashkan K, FitzGerald J, Green A L, Aziz T Z and Brown P 2013 Adaptive deep brain stimulation in advanced Parkinson disease *Ann. Neurol.* **74** 449–57

[2] Habets J G V, Heijmans M, Kuijf M L, Janssen M L F, Temel Y and Kubben P L 2018 An update on adaptive deep brain stimulation in Parkinson's disease *Mov. Disord.* **33** 1834–43

[3] Swann N C, de Hemptinne C, Miocinovic S, Qasim S, Ostrem J L, Galifianakis N B, Luciano M S, Wang S S, Ziman N, Taylor R and Starr P A 2018 Chronic multisite brain recordings from a totally implantable bidirectional neural interface: experience in 5 patients with Parkinson's disease *J. Neurosurg.* **128** 605–16

[4] Stanslaski S, Afshar P, Cong P, Giftakis J, Stypulkowski P, Carlson D, Linde D, Ullestad D, Avestruz A-T and Denison T 2012 Design and validation of a fully implantable, chronic, closed-loop neuromodulation device with concurrent sensing and stimulation *IEEE Trans. Neural Syst. Rehabil. Eng.* **20** 410–21

[5] Zhou A, Johnson B C and Muller R 2018 Toward true closed-loop neuromodulation: artifact-free recording during stimulation *Curr. Opin. Neurobiol.* **50** 119–27

[6] Stanslaski S, Herron J, Chouinard T, Bourget D, Isaacson B, Kremen V, Opri E, Drew W, Brinkmann B H, Gunduz A, Adamski T, Worrell G A and Denison T 2018 A Chronically Implantable Neural Coprocessor for Investigating the Treatment of Neurological Disorders *IEEE Trans. Biomed. Circuits Syst.* **12** 1230–45

[7] Debarros J, Gaignon L, He S, Pogosyan A, Benjaber M, Denison T, Brown P and Tan H 2020 Artefact-free recording of local field potentials with simultaneous stimulation for closed-loop Deep-Brain Stimulation *2020 42nd Annual International Conference of the IEEE Engineering in Medicine Biology Society (EMBC)* pp 3367–70

[8] Neumann W-J, Turner R S, Blankertz B, Mitchell T, Kühn A A and Richardson R M 2019 Toward Electrophysiology-Based Intelligent Adaptive Deep Brain Stimulation for Movement Disorders *Neurotherapeutics* **16** 105–18

[9] Neumann W-J, Memarian Sorkhabi M, Benjaber M, Feldmann L K, Saryyeva A, Krauss J K, Contarino M F, Sieger T, Jech R, Tinkhauser G, Pollo C, Palmisano C, Isaias I U, Cummins D D, Little S J, Starr P A, Kokkinos V, Gerd-Helge S, Herrington T, Brown P, Richardson R M, Kühn A A and Denison T 2021 The sensitivity of ECG contamination to surgical implantation site in brain computer interfaces *Brain Stimul.* **14** 1301–6

[10] Hosain M K, Kouzani A and Tye S 2014 Closed loop deep brain stimulation: an evolving technology *Australas. Phys. Eng. Sci. Med.* **37** 619–34

[11] Dinsmoor D A, Hocken R W, Santa W A, Shah J S, Tyler L and Denison T J 2011 Neurostimulation Design from an Energy and Information Transfer Perspective *Bio-Medical CMOS ICs* ed H-J Yoo and C van Hoof (Boston, MA: Springer US) pp 453–80

[12] Holsheimer J, Dijkstra E A, Demeulemeester H and Nuttin B 2000 Chronaxie calculated from current-duration and voltage-duration data *J. Neurosci. Methods* **97** 45–50

[13] Gimsa J, Habel B, Schreiber U, van Rienen U, Strauss U and Gimsa U 2005 Choosing electrodes for deep brain stimulation experiments--electrochemical considerations *J. Neurosci. Methods* **142** 251–65

[14] Merrill D R, Bikson M and Jefferys J G R 2005 Electrical stimulation of excitable tissue: design of efficacious and safe protocols *J. Neurosci. Methods* **141** 171–98

[15] Powell M P, Anso J, Gilron R, Provenza N R, Allawala A B, Sliva D D, Bijanki K R, Oswalt D, Adkinson J, Pouratian N, Sheth S A, Goodman W K, Jones S R, Starr P A and Borton D A 2021 NeuroDAC: an open-source arbitrary biosignal waveform generator *J. Neural Eng.* **18**
